# Menu labeling and portion size control to improve the out‐of‐home food environment: A scoping review

**DOI:** 10.1002/cesm.12039

**Published:** 2024-01-23

**Authors:** Kathiresan Jeyashree, Rizwan S. Abdulkader, Madhumitha Haridoss, Ranjithkumar Govindaraju, Amanda Brand, Marianne Visser, Sarah Gordon, Hemant Tiwari, T. S. Sumitha, Krupa Chandran, Denny Mabetha, Solange Durão

**Affiliations:** ^1^ Department of Epidemiology and Biostatistics Indian Council of Medical Research, National Institute of Epidemiology Chennai India; ^2^ Department of Cochrane Nutrition Centre for Evidence‐Based Health Care Stellenbosch University Cape Town South Africa; ^3^ Department of Cochrane Nutrition, Health Systems Research Unit South African Medical Research Council Tygerberg South Africa

**Keywords:** malnutrition, menu labeling, portion size control, scoping review

## Abstract

**Background:**

Menu labeling and portion size control interventions may be effective strategies to mitigate the health risks posed by the out‐of‐home food environment. We conducted this scoping review to map the body of evidence (BoE) addressing the effects of menu labeling and portion size control interventions in the out‐of‐home food environment and to summarize the research gaps in this evidence base.

**Methods:**

We searched PubMed, Embase, Epistemonikos, and PROSPERO in phase 1 for systematic reviews (SRs) and PubMed and Embase in phase 2 for primary studies in areas with insufficient SR evidence. We used a comprehensive search strategy without any restrictions on publication date, language, study population characteristics or outcomes. We screened all titles independently and in duplicate. We mapped the number of systematic reviews providing evidence per intervention‐setting combination in a matrix. The gaps in the matrix informed the searches for primary studies in phase 2. For the included SR protocols and primary studies, we charted the population, intervention, comparator, outcome, period, and study design to facilitate their evaluation and inclusion in future evidence syntheses.

**Results:**

We included 69 completed SRs; 37 on menu labeling, 9 on portion size control, and 23 on both. The types of menu labeling interventions studied were quantitative nutrient information (74%), interpretational guidance (48%), or contextual guidance (13%). Most reviews were from the United States, United Kingdom, and Canada. Most SRs included studies in establishments like cafeterias (51%) or restaurants (39%) and measured change in the quantity of food offered/ordered/consumed (96%). Phase 2 search yielded 24 primary studies; 16 experimental, 6 quasi‐experimental, and 2 observational studies.

**Conclusion:**

The BoE on the effectiveness of menu labeling and portion size control is predominantly from the developed world, on nutrient information labeling and reporting impact on consumer food choice. There is a need for studies in the online environment and reporting distal health outcomes.

## 1 INTRODUCTION

Dietary risk factors, such as a high intake of sodium and a low intake of wholegrains, vegetables, and fruits, contribute to 188 million disability‐adjusted life years annually, with cardiovascular diseases being their primary consequence [1]. Although these risk factors are present in the home food environment, they are amplified in the out‐of‐home food environment. Eating out has become more popular in recent years, particularly among young adults [2, 3, 4]. Excess weight gain and adverse cardiometabolic outcomes have been reported in those who eat out frequently [5, 6], with eating out associated with poorer food choices, higher energy and saturated fat intake, and inadequate micronutrient intake [5, 7]. Additionally, increasing portion sizes in the out‐of‐home environment may exacerbate these adverse consequences [8]. People consistently eat more food or drink more nonalcoholic beverages when they are offered larger portions or packages than when offered smaller portions [9]. Growth in portion sizes served out‐of‐home has also been correlated with global increases in obesity rates [7]. In this context, it is essential to help consumers make healthier dietary choices in the out‐of‐home food environment.

Interventions, such as food labeling, which provide nutrition information, and portion size control interventions, which affect perceptual features of food, contribute to consumers making healthier food choices. These interventions may thus be essential policy actions to influence dietary behaviors in the out‐of‐home environment positively. Menu labeling interventions entail the provision of the nutritional content of foods on menus, menu boards, and food item tags at the point‐of‐purchase, traditionally in fast‐food and full‐service restaurants [10], but also increasingly in more unconventional spaces such as on vending machines [11]. Many countries worldwide have implemented, or are starting to implement, menu labeling policies for specific food retailers [12]. Most of these are mandatory policies, but some are voluntary, and they also differ in the level at which they are implemented, that is, regional or national; these aspects may affect uptake. There have also been several calls for menu labeling in online food delivery apps [13], particularly given the rise in the use of this modality to access food from the out‐of‐home environment during the COVID‐19 pandemic; this mode of access is anticipated to become the “new normal” in the postpandemic era [14, 15].

Menu labeling interventions have been evaluated for their effect on a range of outcomes, including food purchases, consumption, as well as physiological and clinical outcomes, with varying results [5, 6]. Some systematic reviews (SRs) on the effectiveness of menu labeling interventions found that they did not lead to any significant change in reported calories, fat, or sodium in food ordered or consumed [16, 17]. On the other hand, one SR found that exposure to nutrition labels positively modified dietary behavior among college students [18]. Two meta‐analyses studying portion size control interventions report that more comprehensive changes to the portion‐size environment and policy are required for interventions like portion size norms, packaging, and serve‐ware size to have sufficient impact [19, 20].

To the best of our knowledge, there has been no comprehensive mapping of the existing evidence base assessing the effectiveness of both menu labeling and portion size control interventions in the out‐of‐home food environment. Thus, this scoping review aimed to identify, quantify, and map the types and sources of evidence available in the peer‐reviewed literature addressing the effects of menu labeling and portion size control interventions in the out‐of‐home food environment and to summarize the research gaps in this evidence base.

## 2 METHODS

We conducted this scoping review according to guidance from the Joanna Briggs Institute on scoping reviews [21] and reported it according to the Preferred Reporting Items for Systematic Reviews and Meta‐Analyses Extension for Scoping Reviews reporting guideline [22]. A proposal detailing the rationale, inclusion and exclusion criteria, search strategy, proposed data charting, and synthesis methods was reviewed and approved by the World Health Organization Department of Nutrition and Food Safety in response to its call for authors (https://www.who.int/news-room/articles-detail/call-for-authors-scoping-review-on-menu-labelling-and-portion-size-control-to-improve-out-of-home-food-environment/) before commencement.

### 2.1 Searching for studies

We implemented a two‐step search to identify relevant studies. In phase 1, we searched Medline PubMed, EMBASE, and Epistemonikos (October 2022) for published SRs and PROSPERO for SR protocols. We used a comprehensive search strategy reviewed by an information specialist (Table S1). There were no restrictions on publication date, language, study population characteristics, or study outcomes. In phase 2, we searched Medline PubMed and Embase (December 2022) for primary studies addressing specific gaps identified through mapping the evidence base covered by the included SRs. This search strategy was informed by the systematic review search strategy but driven by the missing evidence identified by the gap analysis of phase 1 outputs. For example, if there were enough studies reporting on menu labeling and portion size control interventions in establishments like restaurants, search terms for restaurants were not factored into the phase 2 search strategy.

### 2.2 Selecting studies for inclusion

We have presented the eligibility criteria in Box 1. We imported all search results into Covidence (SRs) [23] or Rayyan (primary studies) (https://rayyan.qcri.org/) for deduplication and screening. We screened titles and abstracts, and then the full‐texts of potentially eligible records, independently and in duplicate. We resolved any disagreements by discussion between the two reviewers or with arbitration by a third reviewer to reach a consensus. We also screened the reference lists of all included SRs for additional eligible studies.

BOX 1 **Eligibility criteria for considering studies for inclusion**

**Table:** 

Item	Inclusion criteria	Exclusion criteria
Population	Participants belonging to any age group, gender or ethnicity.	None
Interventions or exposures of interest	*Menu labeling interventions*, defined as policies/interventions that mandate the disclosure of calories or other nutrients of menu items in restaurants or other food retail outlets (e.g., coffee shops, cafeterias, etc.) on menus or menu boards. This could include informative approaches (i.e., provide numeric calorie information only), interpretational approaches (e.g., symbol conveying nutritional quality), or contextual approaches (e.g., a statement about daily caloric needs) [24]. Menu board signposting, shelf labels, labels on foods served, and labeling provided on delivery apps or on delivered food were included. Mandatory and voluntary interventions or policies, as well as those implemented locally (e.g., in a city), regionally or nationally, were eligible for inclusion. A detailed description of the classification of interventions on menu labeling is available in Table S1. *Portion size control* interventions, which included limiting the size (or volume) of food or beverage containers [25] or offering reduced‐sized portions [26].	Food labeling interventions such as ingredient lists, health‐related claims such as those related to allergy or intolerance, front‐of‐package labels. Educational interventions on nutritional labeling Interventions addressing packed beverages and any alcoholic beverages
Comparator	No intervention, or other active interventions, or with “business‐as‐usual.”	None
Outcomes	All outcomes reported in eligible publications to enable mapping of all outcomes in this scoping review.	None
Study design, language, and publication type	Systematic reviews^a^ in any language, published on any date, assessing effects, or associations of menu labeling or portion size control on any outcome, including protocols. Primary studies (interventional and analytical study designs) of interventions/exposures of interest for which no systematic reviews were identified. For example, studies of menu labeling in delivery apps, which may be too recent to have been included in published systematic reviews. Preprint/prepublication records of potentially eligible systematic reviews (at the last search date) were included in the list of ongoing studies and identified as such. Any date or publication and any language.	Scoping reviews, systematic reviews of qualitative studies, and overviews of systematic reviews Observational studies (in primary studies search)

^a^A systematic review will be defined as a review that had predetermined objectives, predetermined criteria for eligibility, searched at least two data sources, of which one needed to be an electronic database, and performed data extraction. Reviews that did not align with this definition will be excluded. Narrative reviews will be excluded.

### 2.3 Data extraction and synthesis

We extracted data into Microsoft Excel using piloted data extraction forms. One reviewer charted the data, with another reviewer cross‐checking a random sample of 20% of included studies. Any disagreements were resolved by discussing or consulting a third reviewer, if necessary. We requested translations from native language‐speaking colleagues for studies that were not in English.

We extracted the following information from included completed SRs: publication year, country where studies were conducted, characteristics of study settings, study population and study designs, details about the interventions, settings, and outcomes reported. Once we charted the data from the included systematic reviews, we created a matrix where eligible primary studies were mapped against the systematic reviews to assess the overlap of studies. We mapped the number of systematic reviews providing evidence per intervention‐setting combination in a matrix. The gaps in the matrix informed the searches for primary studies in phase 2. For the included SR protocols and primary studies, we charted the population, intervention, comparator, outcome, period, and study design to facilitate their evaluation and inclusion in future evidence syntheses.

The methodological quality of included SRs and primary studies was not assessed since this scoping review aimed to map the evidence base and not to determine the effects of interventions or the strength of associations between exposures and outcomes.

We summarized the included studies descriptively, using frequencies and proportions. To describe included menu labeling interventions, we used a framework categorizing the settings where the intervention was implemented (e.g., educational, workplace, online, simulation, or laboratory), the food establishment type where the intervention was implemented (e.g., restaurants, coffee shops, deli counters, vending machines), the format of the labeled menu (e.g., informational, interpretational, or contextual), and the type of parameter that was being described (e.g., calories, macronutrients, micronutrients; Table S2). For portion control intervention, in addition to the settings and establishment, we describe the type of portion size control intervention that was studied (e.g., portion size increase or decrease or tableware size change, change in portion of a particular category of food, for example, reducing portion of fried foods, increasing portion of fruits and vegetables).

## 3 RESULTS

### 3.1 Phase 1: Systematic reviews

#### 3.1.1 Results of the search

**Figure 1:**
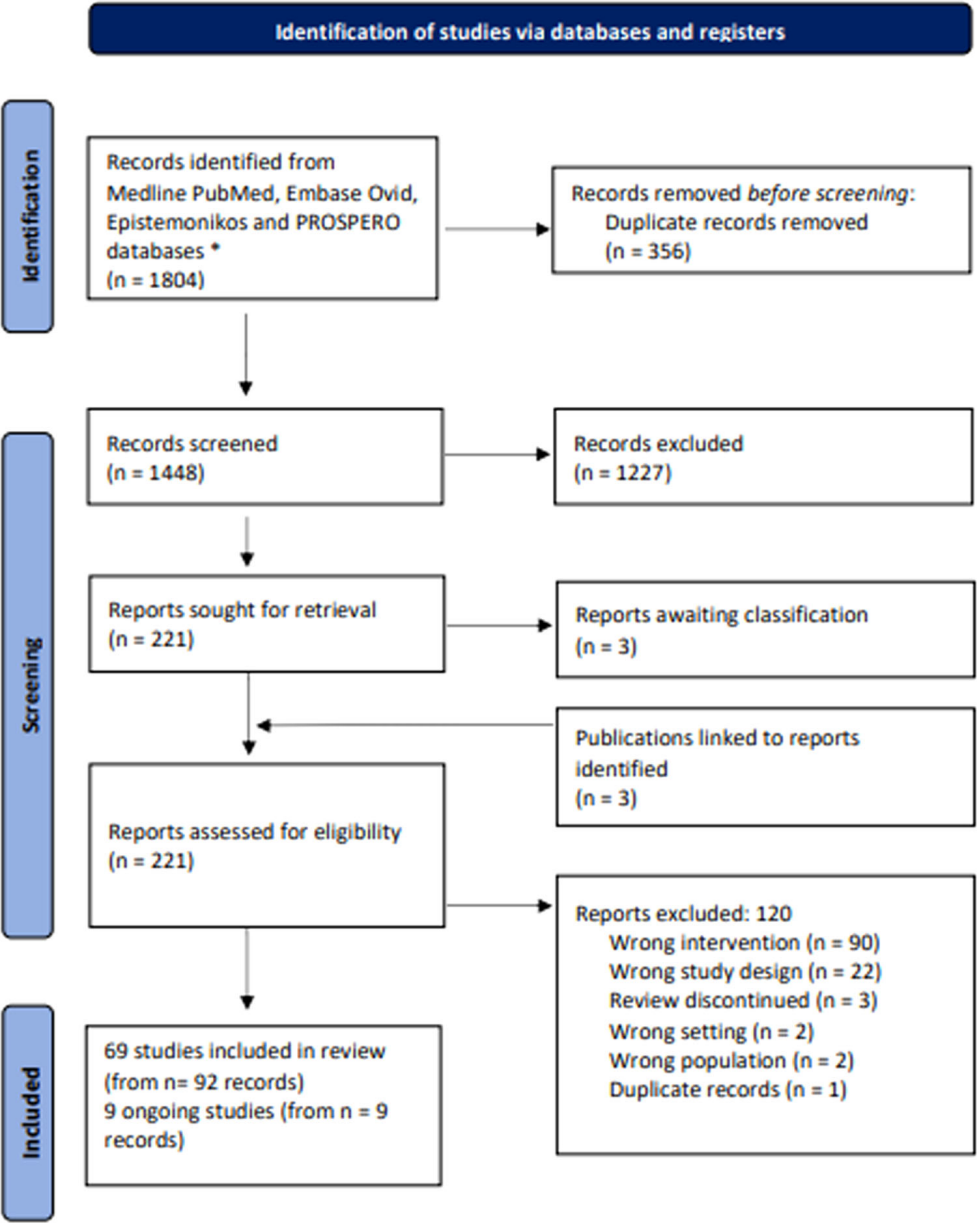
Preferred Reporting Items for Systematic Reviews and Meta‐Analyses diagram for the phase 1 search for eligible systematic reviews. *Records by database: Medline PubMed *n* = 572; Embase Ovid *n* = 685; Epistemonikos *n* = 182; PROSPERO *n* = 365.

Our search for SRs retrieved 1804 records (Figure 1). Following deduplication, we screened the titles and abstracts of 1448 records and the full‐text reports of 221 potentially eligible records against the inclusion criteria. Following the full‐text screening, 69 unique SRs (from 92 records) were included, and 120 records, including 23 protocols, were excluded, with reasons detailed in Figure 1. Three reviews were marked as awaiting classification since their full texts could not be accessed, and we included nine SR protocols as ongoing studies. Details of the reviews, protocols, and primary studies which were included and excluded in this scoping review are presented in Tables S3–S9.

#### 3.1.2 Description of included reviews

The number of reviews on menu labeling and portion size control in out‐of‐home food environment has steadily increased after 2010, with the highest number of studies (*n* = 11) published in 2021 (Figure S1). We did not observe any specific trends or shifts in the focus of interventions over time. However, menu labeling has been consistently studied more than portion control.

A total of 2545 primary studies were cited across 69 completed SRs, with the number of primary studies included in each of the included reviews ranging from 3 to 294 studies. However, not all these primary studies were relevant for this scoping review, and there was some overlap in the primary evidence base across reviews. A total of 496 unique primary studies relevant to our scoping review were included across the 69 completed reviews; these numbers ranged between 1 and 53 studies per review. There was a limited overlap of relevant primary studies across all the included SRs. Most primary studies (*n* = 347; 70%) were cited in a single SR; whereas 15% (*n* = 77) and 6% (*n* = 30) of studies were cited in two and three SRs, respectively. The maximum number of reviews including the same primary study was 14 (20% of included reviews); this occurred for 28 primary studies.

The most common study designs in the included reviews were quasi‐experimental (*n* = 46; 67%) and randomized controlled trials (RCTs) (*n* = 36; 52%). (Table 1) Fewer reviews included cohort (*n* = 8; 12%) or cross‐sectional (*n* = 17; 25%) studies.

**Table 1:** Distribution of study designs of relevant primary studies in the reviews on menu labeling and portion size control in out‐of‐home food environment (*n* = 69).

Study designs in included SRs^a^	Overall (*N* = 69), *N* (%)	Menu labeling (*N* = 37), *N* (%)	Portion size control (*N* = 9), *N* (%)	Both menu labeling and portion size control (*N* = 23), *N* (%)
RCTs	36 (52)	17 (46)	4 (44)	15 (65)
Cohort studies	8 (12)	4 (11)	2 (22)	2 (8.7)
Cross‐sectional studies	17 (25)	11 (30)	0 (0)	5 (22)
Quasi‐experimental studies	46 (67)	28 (76)	5 (56)	13 (57)
Others^b^	10 (14)	6 (16)	0 (0)	4 (17)

Abbreviation: RCT, randomized control trial.

^a^ Numbers represent reviews that have included at least one study of a particular design type.

^b^ Case‐control design, policy analysis, mixed‐methods study, individual‐level microsimulation model, and multistate Markov modeling over a lifetime time horizon.

The studies included in the eligible reviews were mainly from the United States (*n* = 58; 84.1%), followed by the United Kingdom (*n* = 18; 26.1%) and Canada (*n* = 16; 23.2%) (Figure S2). More than a third of the reviews included studies conducted in populations of all ages; 15 reviews included only adults, 9 included only children or adolescents, and 18 (26%) reviews did not specify the age groups included.

**Figure 2:**
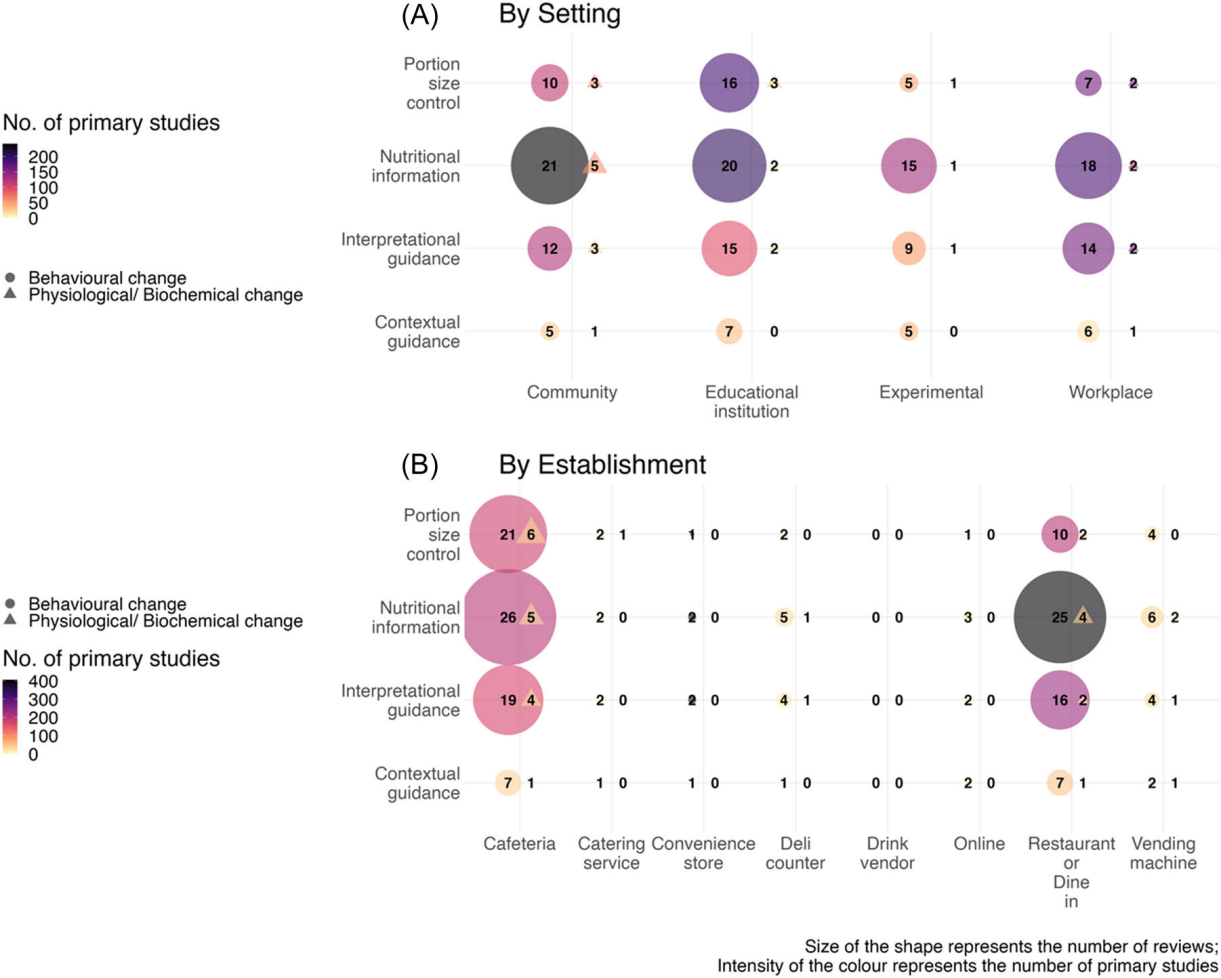
Evidence gap map showing the availability of evidence in terms of number of reviews and primary studies on the effect of different interventions on behavioral outcomes and physiological/biochemical outcomes: (A) by setting and (B) by establishment.

Of the 69 completed reviews included, 37 addressed menu labeling, 9 addressed portion size control, and 23 addressed both interventions. Most reviews addressing menu labeling interventions included studies assessing the provision of nutrient information (*n* = 51; 74%), followed by those providing interpretational guidance (*n* = 33; 48%) (see Box 2 for examples). Few reviews included studies assessing menu labels in the form of contextual guidance (*n* = 9; 13%). Portion size control interventions included studies evaluating the provision of smaller portion sizes of unhealthy food options like fried foods (*n* = 10) or larger portion sizes of healthy food options like salads (*n* = 4) or both (*n* = 4). Six reviews evaluated the effect of changing the size of portions served, and three reviews studied the impact of changes in tableware size. Overall, the included reviews included studies distributed evenly across educational (*n* = 30; 43%), workplace (*n* = 22; 32%), community (*n* = 26; 38%), and experimental (*n* = 19; 28%) settings (Table 2). Reviews focusing on portion size control and those assessing menu labeling and portion size control were conducted more often in educational settings (5/9; 56% and 12/23; 52%, respectively). Reviews on menu labeling included studies primarily conducted in restaurants (18/37; 49%) and cafeterias (14/37; 38%). Very few reviews included studies focused on catering services, convenience stores, deli counters, or online application settings (Figure 2). Studies included in reviews on portion size control (6/9; 67%), and reviews studying both menu labeling and portion size control (15/23; 65%), were mainly carried out in cafeterias (Table 2). Three reviews on menu labeling and one on both menu labeling and portion size control focused on the online food environment. There were no studies which tested these interventions among drink vendors.

BOX 2 **Formats of menu labeling interventions in the out‐of‐home environment explored in the included reviews**

**Table:** 

Format of menu labeling	Number (%) of studies, *N =* 69	Examples
Nutrient information	51 (74%)	Calorie labeling, kilojoule labeling, energy labeling, sodium, sugar content, and low‐fat labeling.
Interpretational guidance	33 (48%)	Traffic lights, color‐coded stickers green and red to indicate healthy or unhealthy food, brightly colored “0 calories, 0 carbs” labels, heart‐healthy logos, “healthy dining” labels based on Food and Drug Administration criteria, obvious graphic salt warnings, heart/apple symbol indicating healthy choice, physical activity calorie equivalent, star rating, and certification logos.
Contextual guidance	9 (13%)	Recommended daily caloric intakes, statements related to meal components selected, such as “your meal does not look like a balanced meal.”

*Note*: Percentages within parentheses do not add up to 100% as some of the reviews included studies assessing more than one type of menu labeling intervention.

**Table 2:** Distribution of study settings and establishments in the reviews on menu labeling and portion size control in out‐of‐home food environment (*n* = 69).

Setting	Overall (*N* = 69), *N* (%)	Menu labeling (*N =* 37), *N* (%)	Portion size control (*N* = 9), *N* (%)	Both menu labeling and portion size control (*N* = 23), *N* (%)
By setting				
Education	30 (43)	13 (35)	5 (56)	12 (52)
Workplace	22 (32)	14 (38)	1 (11)	7 (30)
Community	26 (38)	18 (49)	2 (22)	6 (26)
Experimental	19 (28)	15 (41)	1 (11)	3 (13)
By establishment				
Restaurant	27 (39)	18 (49)	1 (11)	8 (35)
Cafeteria	35 (51)	14 (38)	6 (67)	15 (65)
Catering services	3 (4.3)	1 (2.7)	1 (11)	1 (4.3)
Vending machine	9 (13)	5 (14)	1 (11)	3 (13)
Deli counter	5 (7.2)	3 (8.1)	0 (0)	2 (8.7)
Convenience store	2 (2.9)	1 (2.7)	0 (0)	1 (4.3)
Online apps	4 (5.8)	3 (8.1)	0 (0)	1 (4.3)
Other	11 (16)	9 (24)	1 (11)	1 (4.3)

Reviews included primary studies that either compared the effect of the intervention across two study groups or studied a single group before and after receiving the intervention of interest. Studies comparing two groups included those that compared one group who received the intervention of interest with another group who also received an active intervention (*n* = 21; 30%), such as other menu labeling or portion size control interventions, educational or pricing interventions which did not receive any intervention (*n* = 33; 69%). Twenty‐seven (39%) reviews included studies that compared a single group before and after giving the intervention.

Most of the included reviews (*n* = 66; 96%) reported changes in the quantity of food offered, ordered or consumed (Box 3). Few reviews reported changes in physiological/biochemical parameters (*n* = 15; 22%) such as body mass index (BMI), body weight, and waist‐to‐hip ratio.

BOX 3 **Examples of outcomes studied in the reviews on menu labeling and portion size control in out‐of‐home food environment (*n* = 69)**

**Table:** 

Type of outcome	Examples of outcomes reported in included reviews
Change in food ordered/consumed	Energy intake Dietary intake and/or purchases (e.g., intake of fruit and vegetables, sugary drinks, energy‐dense foods, fast foods) Calories offered (changes in restaurant offerings) Sales of healthier milk, refried beans, cream cheese, and peanut butter Change in sodium consumption and purchase Sales records of the number of calories purchased per day Self‐reported frequency of healthy food requests Increased percentage of healthy dishes consumption Proportion of all meals sold that were a smaller portion Change in consumption of a food item when offered a larger portion size
Change in physiological/biochemical parameters	Change in risk factors (obesity, overweight, weight gain, control, maintenance, BMI, body weight, adiposity, fat mass, skinfold thickness, waist circumference, waist‐hip ratio), cholesterol
Other outcomes	Perceptions about the interventions Proportion noticing the implemented intervention (e.g., proportion of participants noticing and using the labels on the menu) Food wastage Consumer satisfaction DALYs and QALYs Incremental cost‐effectiveness ratios of the menu labeling intervention per unit BMI reduced

Abbreviations: BMI, body mass index; DALY, disability‐adjusted life year; QALY, quality‐adjusted life year.

Of the 69 included reviews, 48 (70%) included a conflict‐of‐interest statement, of whom 42 (88%) had declared no conflict of interests, and in six reviews [9, 27, 28, 29, 30, 31], a conflict statement was reported. Reported conflict statements included receiving funding from one of the private consumer products companies, having chaired groups that encourage the adoption of energy labeling on menus, holding a position paid by one of the funding agencies, or being part of the author group of trials included in the review.

Forty‐six (67%) of the 69 reviews reported their source of funding, 19 (28%) reviews did not report on funding, and 4 (5%) reviews reported not receiving any funding support for the review.

#### 3.1.3 Description of ongoing reviews

All nine included SR protocols were registered between 2019 and 2022 and aimed to include experimental, quasi‐experimental, or observational studies. All of these intended to report changes in food purchase, and in the consumption of healthy foods and drinks, except one protocol which intended to report on the “population prevalence of nutrition/non‐communicable diseases (NCDs).”

### 3.2 Phase 2: Primary studies

#### 3.2.1 Results of the search

We first charted the gaps in the evidence for menu labeling and portion size control interventions in terms of the patient, intervention, comparison, and outcomes addressed by the included reviews (Figure 2). We identified gaps for the menu labeling interventions in drink vendor settings and for portion size control interventions in the following settings: catering services, deli counters, convenience stores, drink vendors, and online. We carried out the phase 2 search for primary studies to address these gaps.

**Figure 3:**
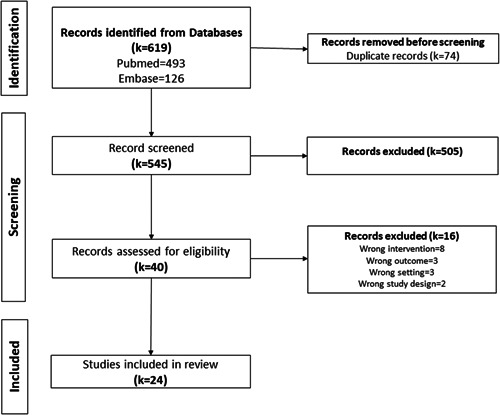
Preferred Reporting Items for Systematic Reviews and Meta‐Analyses diagram for the phase 2 search for eligible primary studies.

The search retrieved 545 deduplicated records, the titles and abstracts of which were screened in duplicate. After screening 40 potentially eligible full‐texts, 24 primary studies were included, and 16 records were excluded (Figure 3).

#### 3.2.2 Description of included primary studies

All of the 24 primary studies included had studied portion size control interventions. Sixteen were experimental study designs, including RCTs and cross‐over trials, six were quasi‐experimental studies, and only two were observational studies (Table S8). Studies were conducted in various settings, including educational (*n* = 11), community setting (*n* = 11), laboratory (*n* = 1), and workplace (*n* = 1) settings. Seven studies had exclusively included women as their study population, one of them specifically focusing on overweight and obese women.

## 4 DISCUSSION

Our scoping review included 69 completed SRs and 9 SR protocols on menu labeling and portion size control interventions in phase 1 and 24 primary studies on portion size control interventions in phase 2. There was little overlap of included primary studies across included SRs, suggesting heterogeneity within their scope or methodological approaches. Studies included in these reviews were mostly conducted in apparently healthy populations in a workplace, educational, community, and experimental setting, and predominantly in establishments, such as restaurants and cafeterias. Reviews mainly explored the effect of interventions on changes in the quantity of food/specific food item/specific nutrient offered, purchased, consumed or wasted, and on physiological, biochemical, and anthropometric outcomes.

Overall, the evidence base on the effect of portion size control interventions in the out‐of‐home food environment is limited, especially in settings like the workplace and online environments. Following a gap analysis, the phase 2 search yielded 24 primary studies that could potentially be included in future SRs evaluating portion control interventions. As far as reviews on menu labeling interventions are concerned, not many reviews reported a head‐to‐head comparison between different types of menu labeling interventions but have rather restricted to before–after comparisons or comparisons with no intervention.

Most of the included reviews did not include studies conducted in developing countries. This geographical distribution of studies is probably representative of the lag in the epidemiological transition between developed and developing countries. Developed countries, which have been facing the brunt of surges in NCDs and a relatively smaller burden of infectious diseases, are also the areas from where research on nutrition and NCDs is plenty. There is a negligible representation of African, Asian and South American regions, which points to the lack of primary research output from these regions on menu labeling and portion size control interventions. There is a need to put these interventions on the research priority lists of these regions as many developing countries or regions are already facing a very high burden of NCDs.

Few included reviews studied the effect of menu labeling and portion size interventions in the out‐of‐home food environment among specific risk factors or disease groups, for example, obese individuals or people with diabetes mellitus or other NCDs or risk factor populations. Such focused studies will help identify the priority populations to implement these interventions and if there is a differential effect of these interventions across these special study populations compared to apparently healthy populations.

Few reviews assessed the online setting. Since the online setting is a relatively recent development, more studies may emerge in the future, offering insight into the effect of menu labeling and portion size control interventions in these settings.

Most reviews reported immediate and short‐term health outcomes such as lowered consumption of calories or unhealthy food, and very few looked at long‐term sustained health benefits. There is a need for evidence on the long‐term effects of these interventions, such as changes in biochemical (e.g., blood glucose levels, cholesterol levels) and anthropometric outcomes (e.g., weight, BMI). Such studies can be resource‐intensive and methodologically challenging, given the longer follow‐up and multiple confounding factors that also influence these parameters besides the intervention. But, they are vital to understanding whether these interventions have any significant long‐term impact at all. There have been very few reviews studying policies and programs implementing these interventions exclusively or as a part of a larger multicomponent intervention. More such studies assessing the impact of these policies in real‐world settings are required, given their ability also to assess the feasibility, sustainability and cost‐effectiveness of these interventions.

### 4.1 Strengths and limitations

Our scoping review mapped the breadth of the evidence base available on menu labeling and portion size control interventions in out‐of‐home food environments. Our search strategy was refined by an information specialist, and it was comprehensive—covering three databases and one registry (PROSPERO) to ensure no relevant studies were missed (in phase 1). We conducted both title/abstract screening as well as full‐text screening in duplicate to minimize error. We conducted rigorous quality checks at each level of screening and data extraction for phases one and two of our review, which should minimize bias from being introduced. Our gap analysis and informed phase 2 search ensured that we did not miss primary studies addressing interventions conducted in specific settings, for example, in the online setting, which has more recently started being studied.

We have not analyzed the barriers and facilitators of the implementation of these interventions; this would have immense relevance to policymakers. We have not provided a granular analysis of the included primary studies as they were the results of a very specific search strategy to assess the availability of evidence for the interventions in certain settings.

## 5 CONCLUSION

SRs with an exclusive focus on the effect of menu labeling and portion size control interventions in the out‐of‐home food environment on behavioral and health outcomes are required. More primary studies are required from the developing and underdeveloped nations to understand the evidence base on these interventions from these regions. There is a specific need for primary studies on the effect of portion size control interventions in different out‐of‐home environment settings. For both interventions, there is a need for more research to study their impact on a wider range of outcomes which are of relevance to all stakeholders to aid in effective implementation. Costing studies on both interventions are required.

## AUTHOR CONTRIBUTIONS

**Kathiresan Jeyashree**: Conceptualization; data curation; formal analysis; funding acquisition; methodology; project administration; resources; software; supervision; validation; writing—original draft; writing—review and editing. **Rizwan S. Abdulkader**: Data curation; formal analysis; methodology; validation; visualization; writing—review and editing. **Madhumitha Haridoss**: Data curation; resources; software; visualization; writing—review and editing. **Ranjithkumar Govindaraju**: Data curation; resources; software; visualization; writing—review and editing. **Amanda Brand**: Data curation; software; visualization; writing—review and editing. **Marianne Visser**: Data curation; supervision; visualization; writing—review and editing. **Sarah Gordon**: Resources; software; visualization; writing—review and editing. **Hemant Tiwari**: Resources; software; visualization. **T. S. Sumitha**: Resources; software; visualization. **Krupa Chandran**: Resources; software; visualization. **Denny Mabetha**: Resources; software; visualization. **Solange Durão**: Data curation; formal analysis; resources; software; validation; writing—review and editing.

## CONFLICT OF INTEREST STATEMENT

The authors declare no conflict of interest.

## PEER REVIEW

The peer review history for this article is available at https://www.webofscience.com/api/gateway/wos/peer-review/10.1002/cesm.12039.

## Supporting information

Supporting information.

## Data Availability

The data that support the findings of this study are available from the corresponding author upon reasonable request.
