# Supplementary material for: Menu labeling and portion size control to improve the out‐of‐home food environment: A scoping review
Source: Cochrane Evid Synth Methods. 2024 Jan 23;2(1):e12039. doi: 10.1002/cesm.12039 (PMC11795951; doi:10.1002/cesm.12039)

Supplementary Appendix

Table of Contents

[Table. S1: Search strategy for Medline PubMed 2](#_Toc136941177)

[Table. S2: Classification/description of interventions 5](#_Toc136941178)

[Table. S3: Characteristics of included studies - completed reviews (n=69) 7](#_Toc136941179)

[Table. S4: Characteristics of excluded studies - completed reviews (n=92) 27](#_Toc136941180)

[Table. S5: Characteristics of studies awaiting classification (n=3) 32](#_Toc136941181)

[Table. S6: Characteristics of included protocols (n=9) 33](#_Toc136941182)

[Table. S7: Characteristics of excluded protocols (n=23) 36](#_Toc136941183)

[Table. S8- Characteristics of included primary studies (n=24) 38](#_Toc136941184)

[Table. S9- Characteristics of excluded primary studies (n=16) 42](#_Toc136941185)

[Figure. S1 Time trends of the included reviews on menu labeling and portion size control (n = 69) 43](#_Toc136941186)

[Figure. S2. Geographical distribution of the included reviews on menu labeling and portion size control in out-of-home food environment (n=69) 44](#_Toc136941187)

## Table. S1: Search strategy for Medline PubMed

**Phase one: Search for systematic reviews**

| **Search number** | **Query** | **Results** |
| --- | --- | --- |
| 21 | (#16 OR #17 OR #18 OR #19 OR #20) AND #1 | 466 |
| 20 | #15 AND #14 | 100 |
| 19 | #15 AND #13 | 1031 |
| 18 | #15 AND #12 | 231 |
| 17 | #15 AND #11 | 454 |
| 16 | #15 AND #10 | 12073 |
| 15 | #8 OR #9 | 44787 |
| 14 | #2 AND #3 AND #7 | 661 |
| 13 | #2 AND #7 | 15223 |
| 12 | #2 AND #6 AND #7 | 1345 |
| 11 | #2 AND #3 AND #6 | 2793 |
| 10 | #2 AND #5 | 1884957 |
| 9 | “food environment”[Title/Abstract] OR “food delivery”[Title/Abstract] OR “meal delivery”[Title/Abstract] OR “delivered meals”[Title/Abstract] OR “take away”[Title/Abstract] OR takeaway[Title/Abstract] OR “food ordering”[Title/Abstract] OR “digital food environment”[Title/Abstract] OR “meal apps”[Title/Abstract] OR “meal app”[Title/Abstract] OR “delivery app”[Title/Abstract] OR “delivery apps”[Title/Abstract] | 4717 |
| 8 | Restaurants[MeSH] OR restaurant*[Title/Abstract] OR “fast food”[Title/Abstract] OR fast foods[MeSH] OR “food service*”[Title/Abstract] OR “food establishment”[Title/Abstract] OR “food stall*”[Title/Abstract] OR dine[Title/Abstract] OR dining[Title/Abstract] OR diner*[Title/Abstract] OR café*[Title/Abstract] OR cafeteria*[Title/Abstract] OR canteen*[Title/Abstract] OR hotel*[Title/Abstract] OR eatery[Title/Abstract] OR eateries[Title/Abstract] OR “eating place*”[Title/Abstract] OR bistro*[Title/Abstract] OR buffet[Title/Abstract] OR catering[Title/Abstract] OR “coffee shop*”[Title/Abstract] OR bars[Title/Abstract] OR “street food”[Title/Abstract] OR streetfood OR “food outlets”[Title/Abstract] OR “convenience food*”[Title/Abstract] OR deli[Title/Abstract] OR “vending machine*”[Title/Abstract] | 41221 |
| 7 | “portion size”[MeSH Terms] OR “portion size”[Title/Abstract] OR “serving size”[MeSH Terms] OR “portion control”[Title/Abstract] OR “serving*”[Title/Abstract] OR “helpings”[Title/Abstract] OR plateful[Title/Abstract] OR bowlful[Title/Abstract] | 53276 |
| 6 | policy[Title/Abstract] OR policies[Title/Abstract] OR legislation[Title/Abstract] OR law[Title/Abstract] OR “Nutrition Policy”[Mesh] | 461470 |
| 5 | #3 OR #4 | 10727938 |
| 4 | educat*[Title/Abstract] OR symbol*[Title/Abstract] OR sign*[Title/Abstract] OR content*[Title/Abstract] OR ticket*[Title/Abstract] OR tags[Title/Abstract] OR information[Title/Abstract] OR sticker*[Title/Abstract] OR nudg*[Title/Abstract] OR Nutripoints[Title/Abstract] OR “guiding stars”[Title/Abstract] OR “Nutrition iQ”[Title/Abstract] OR nutriscore[Title/Abstract] OR nutristar[Title/Abstract] OR “traffic light*”[Title/Abstract] OR choice[Title/Abstract] OR choice[Title/Abstract] OR “rating system”[Title/Abstract] | 10374532 |
| 3 | “Food Labeling”[Mesh] OR label*[Title/Abstract] | 589620 |
| 2 | “Diet, Food, and Nutrition”[Mesh] OR nutrition*[Title/Abstract] OR nutrient*[Title/Abstract] OR food[Title/Abstract] OR diet*[Title/Abstract] OR healthier[Title/Abstract] OR healthy[Title/Abstract] OR energy[Title/Abstract] OR calori*[Title/Abstract] OR kilojoule*[Title/Abstract] OR sugar[Title/Abstract] OR salt[Title/Abstract] OR sodium[Title/Abstract] OR fat[Title/Abstract] OR “saturated fat”[Title/Abstract] OR “Nutritive Value”[Mesh] OR “guideline daily amount*”[Title/Abstract] OR “recommended daily amount*”[Title/Abstract] OR “nutrient reference value*”[Title/Abstract] OR “nutrient daily value*”[Title/Abstract] OR “nutrient daily intake*” OR “dietary reference intake*”[Title/Abstract] OR “Recommended Dietary Allowances”[Mesh] OR “Recommended Dietary Allowances”[Title/Abstract] | 4164310 |
| 1 | “Meta-Analysis”[Publication Type] OR “Meta-Analysis as Topic”[MeSH Terms] OR “Meta- Analysis”[Title/Abstract] OR “Systematic Review”[Publication Type] OR “Systematic Reviews as Topic”[MeSH Terms] OR “Systematic Review”[Title/Abstract] OR “search*”[Title/Abstract] | 771227 |

**Phase two: Search for primary studies**

| Search number | Query | Results |
| --- | --- | --- |
| 7 | #5 AND #6 | 493 |
| 6 | #3 OR #4 | 14,659 |
| 5 | (“Diet, Food, and Nutrition”[Mesh] OR nutria*[Title/Abstract] OR food[Title/Abstract] OR diet*[Title/Abstract] OR health*[Title/Abstract] OR healthy[Title/Abstract] OR energy[Title/Abstract] OR calori*[Title/Abstract] OR kilojoule*[Title/Abstract] OR sugar*[Title/Abstract] OR salt[Title/Abstract] OR sodium[Title/Abstract] OR fat*[Title/Abstract] OR “saturated fat”[Title/Abstract] OR “Nutritive Value”[Mesh] OR “guideline daily amount*”[Title/Abstract] OR “recommended daily amount*”[Title/Abstract] OR “Recommended Dietary Allowances”[Mesh] OR “Recommended Dietary Allowances”[Title/Abstract]) AND (“portion size”[MeSH] OR “portion size*”[Title/Abstract] OR “serving size”[MeSH] OR “portion control”[Title/Abstract] OR serving*[Title/Abstract] OR “helpings”[Title/Abstract] OR plate*[Title/Abstract] OR bowl*[Title/Abstract]) | 1,10,820 |
| 4 | “food environment”[Title/Abstract] OR “food delivery”[Title/Abstract] OR “meal delivery”[Title/Abstract] OR “delivered meals”[Title/Abstract] OR “take away*”[Title/Abstract] OR takeaway*[Title/Abstract] OR “food ordering”[Title/Abstract] OR “ordering of food”[Title/Abstract] OR “ordered food”[Title/Abstract] OR “digital food environment”[Title/Abstract] OR “online food environment”[Title/Abstract] OR “meal apps”[Title/Abstract] OR “meal app”[Title/Abstract] OR “delivery app”[Title/Abstract] OR “delivery apps”[Title/Abstract] | 5,222 |
| 3 | hotel*[Title/Abstract] OR buffet[Title/Abstract] OR catering[Title/Abstract] OR “convenience food*”[Title/Abstract] OR deli[Title/Abstract] OR “convenience store*”[Title/Abstract] OR “drink vendor*” [Title/Abstract] | 9,735 |
| 2 | “portion size”[MeSH] OR “portion size*”[Title/Abstract] OR “serving size”[MeSH] OR “portion control”[Title/Abstract] OR serving*[Title/Abstract] OR “helpings”[Title/Abstract] OR plate*[Title/Abstract] OR bowl*[Title/Abstract] | 5,47,719 |
| 1 | “Diet, Food, and Nutrition”[Mesh] OR nutria*[Title/Abstract] OR food[Title/Abstract] OR diet*[Title/Abstract] OR health*[Title/Abstract] OR healthy[Title/Abstract] OR energy[Title/Abstract] OR calori*[Title/Abstract] OR kilojoule*[Title/Abstract] OR sugar*[Title/Abstract] OR salt[Title/Abstract] OR sodium[Title/Abstract] OR fat*[Title/Abstract] OR “saturated fat”[Title/Abstract] OR “Nutritive Value”[Mesh] OR “guideline daily amount*”[Title/Abstract] OR “recommended daily amount*”[Title/Abstract] OR “Recommended Dietary Allowances”[Mesh] OR “Recommended Dietary Allowances”[Title/Abstract] | 64,55,987 |

## Table. S2: Classification/description of interventions

*Menu labelling – provides information regarding nutrient content of menu items in restaurants or other food retail establishments on menus, menu boards, etc.*

**Setting:** Real world vs experimental setting (yes/no)

**Food establishment type**

- Restaurants
- Fast food restaurants
- coffee shops
- catering services
- vending machines
- cafeterias
- canteen
- drink vendors
- Deli counter in retail establishments
- other

**Where**

- paper menus
- table menus
- electronic menus
- menu boards
- drive-through menus
- menu applications (phone and web)
- advertisements or promotional flyers
- cashier desks
- convenience stores
- drink vendors
- other

**What**  is labelled for menu items:

- calories
- macronutrients: carbohydrates, protein, fat
- sodium
- total sugar
- additionally: a statement related to average daily intake or physical activity equivalent

**Format of labelling:**

| Menu labelling* | | |
| --- | --- | --- |
| Nutrient information | Interpretational guidance | Contextual guidance |
| List amount of calories, micronutrients, macronutrients, sodium, total sugar, caffeine | Symbol conveying nutritional quality (e.g. Calorie/multi-nutrient traffic light) | Statement about daily caloric needs |
|  | Miles/km of walking equivalent | %DI |
|  | Distance of walking equivalent |  |

*multiple approaches could be used

## Table. S3: Characteristics of included studies - completed reviews (n=69)

| **S.No** | **Study ID** | **Title** | **Aim** | **Population** | **Intervention** | **Comparator Group** | **Outcome** | **Eligible Study Design** |
| --- | --- | --- | --- | --- | --- | --- | --- | --- |
| 1 | Al-Khudairy 2019 ^32^ | Choice architecture interventions to improve diet and/or dietary behaviour by healthcare staff in high-income countries: a systematic review | To inform the evidence base on choice architecture interventions to increase healthier purchasing and/ or consumption of food and drink by NHS staff. | Adults | 1. Calorie labelling 2. Traffic light 3. Portion size (smaller portion (i.e., about two thirds of the size of the existing portion) was offered in addition to the existing portion) | No intervention | Dietary (food and/or drink) purchasing (e.g., sales data; receipts analysis) | NR |
| 2 | Atanasova 2022 ^33^ | The impact of the consumer and neighbourhood food environment on dietary intake and obesity-related outcomes: A systematic review of causal impact studies | To systematically review and appraise the evidence on the causal impact studies on the relationship between the built food environment on both dietary intake and obesity-related anthropometric outcomes | All age groups | Calorie labelling and traffic light labelling | NR | Dietary Intake and Purchases (intake of fruit and vegetables (FV), sugary drinks, energy-dense foods, fast foods). 2. Obesity related outcome (e.g., BMI, Weight) | 1. Randomized controlled Trial 2. Quasi experimental study |
| 3 | Beauchamp 2013 ^34^ | The effect of obesity prevention interventions according to socioeconomic position: a systematic review | To identify public health interventions for the primary prevention of obesity that report their effect on anthropometric outcomes by socioeconomic strata, to summarize the effectiveness of these interventions across the socioeconomic spectrum and to identify common attributes of interventions that may be most likely to benefit all SEP groups. | Adults | Calorie labelling (Physical activity to reduce calories) | NR | Change in risk factors (Obesity, overweight, weight (gain, control, maintenance) body mass index, body weight, adiposity, anthropometric, fat mass, skinfold thickness, waist circumference, waist hip ratio) | Quasi experimental study |
| 4 | Bennett 2018 ^26^ | Methods for Evaluating Natural Experiments in Obesity - A Systematic Review | To identify studies reporting the effects of programs, policies, or built environment changes on obesity prevention and control, and to describe their methods to better understand the population-based data sources, data linkages, and methodological and analytic approaches. | All age groups | Calorie labelling | NR | Obesity outcomes for adults (body weight or BMI) or children (BMI z-score or percentile); obesity-related individual health behaviours (dietary and physical activity) | Quasi experimental study |
| 5 | Blake 2019 ^35^ | Investigating business outcomes of healthy food retail strategies: A systematic scoping review | (i) Synthesize the evidence to date on the effect of school food service initiatives on business outcomes, including commercial viability, stakeholder perceptions and customer perceptions, and initiative maintenance and scale-up; (ii) examine whether business outcomes vary by the type or duration of initiative; and (iii) explore the relationship between business outcomes and health behaviour outcomes of healthy food retail initiative in schools. | All age groups | 1. Menu Labelling 2. Portion size control | Comparator group with other intervention/before or after | Commercial viability, Stakeholder perceptions, Customer perceptions, Customer perceptions, Initiative maintenance and scaleup. | 1. Randomized controlled Trial 2. Quasi experimental study 3. study- pure quantitative; 4. studies- pure qualitative- post only no controlled |
| 6 | Bleich 2017 ^12^ | A Systematic Review of Calorie Labeling and Modified Calorie Labeling Interventions: Impact on Consumer and Restaurant Behavior | Consumer responses to calorie information alone or compared to modified calorie information and (2) changes in restaurant offerings following or in advance of menu labelling implementation | All age groups | Calorie labelling on menus | NR | calories ordered, consumed, or available for purchase on restaurant menus | 1. Randomized controlled Trial 2. Quasi experimental study 3. Cross sectional study |
| 7 | Cadario 2018 ^36^ | Which healthy eating nudges work best? A Meta-Analysis of Field Experiments | Effectiveness in field settings of seven healthy eating nudges (1) cognitively oriented, such as “descriptive nutritional labelling,” “evaluative nutritional labelling,” or “visibility enhancements”; (2) affectively oriented, such as “hedonic enhancements or “healthy eating calls”; or (3) behaviourally oriented, such as “convenience enhancements” or “size enhancements.” | All age groups | 1. Calorie/Nutritional labelling 2. Green stickers, smileys, "heart healthy" logos, red stickers next to unhealthier options 3. Size enhancements (Larger plates for healthier options, smaller plates or portions for unhealthy options) | NR | food selection or consumption (either in weight or energy). | Quasi experimental study |
| 8 | Cameron 2016 ^27^ | A Systematic Review of the Effectiveness of Supermarket-Based Interventions Involving Product, Promotion, or Place on the Healthiness of Consumer Purchases | This study aimed to conduct a systematic review of the effectiveness of supermarket-based interventions involving product, promotion or place, on the healthiness of consumer purchases. | NR | Nutritional Labelling (Three labels: 1) Low fat 2) Low calorie, low fat, no trans-fat 3) Low Fat endorsed by the FDA) | NR | Sales of healthier milk, refried beans, cream cheese and peanut butter increased, but healthier mayonnaise and salad dressing decreased | 1. Randomized controlled Trial 2. Quasi experimental study 3. Cohort study |
| 9 | Cantu-Jungles 2017 ^37^ | A Meta-Analysis to Determine the Impact of Restaurant Menu Labeling on Calories and Nutrients (Ordered or Consumed) in U.S. Adults | To assess the effect of restaurant menu labelling on caloric choice or intake of carbohydrate, total fat, saturated fat, and sodium choice compared with before menu labelling or compared with a control group, in an away-from-home setting in the U.S. adult population | Adults | Nutritional labelling | No intervention, before/ after | Estimates of energy, carbohydrates, total fat, saturated fat, and sodium purchased or consumed | 1.Randomized controlled Trial (RCT) 2. Quasi-Randomized controlled Trial (Quasi-RCT) 3. Controlled Before-and-After (CBA) study 4. Interrupted Time Series (ITS) Study 5. Cross-Sectional Study |
| 10 | Carins 2021 ^38^ | Creating supportive eating places: a systematic review of food service initiatives | To examine initiatives deployed in the food service sector to positively influence consumer behaviour, consider the impact these initiatives have had on consumer and public health and to highlight implications of findings for the development and implementation of strategies to improve consumer health | NR | 1. Calorie labelling (Certification logos on food items) 2. Portion size ("Three schemes (19%; Healthier Catering Commitment, Choose Health LA, Public Health Responsibility Deal) focussed on portion size offering smaller options in addition to regular-sized options") | NR | NR | 1. Cohort study 2. Cross sectional study 3. Quasi experimental study 4. Case controlled study 5. Case study 6. Qualitative study |
| 11 | Carter 2018 ^39^ | Information-based cues at point of choice to change selection and consumption of food, alcohol and tobacco products: a systematic review | To estimate the effect of information-based cues on selection and consumption of food, alcohol and tobacco products | NR | 1.Menu Labelling (Signs consisting of indication that it was a healthy choice, green leaf logo indicating healthy options) | other intervention | NR | 1. Randomised controlled trials, or cluster-randomised trials. 2. Non-randomised controlled trials |
| 12 | Cesareo 2022 ^40^ | The effectiveness of nudging interventions to promote healthy eating choices: A systematic review and an intervention among Italian university students | A systematic review of the international literature related to nudging interventions carried out in university cafeterias. to check whether studies were already carried out in Italy, and summarizing what has already been done on the topic in order to use this evidence to develop new intervention(s) | NR | 1.Calorie/Nutritional labelling 2. Traffic light | Between / within cafeteria | NR | 1.Before after study 2. Within and between comparison |
| 13 | Christoforou 2016 ^41^ | State-level and community-level salt reduction initiatives: a systematic review of global programmes and their impact | Achieving global salt reduction targets, the study aims to systematically review such interventions and document reported impact where programmes have been evaluated. | NR | 1.Menu Labelling (Sodium content labelling) | NR | NR | NR |
| 14 | Cohen 2021 ^42^ | Strategies to improve school meal consumption: A systematic review | To systematically review the evidence regarding the impact of various strategies to improve school meal consumption | Children | Portion size (There was a 50% increase in portion sizes for fruits/vegetables; An increase in the portion size of the entrée (i.e., the number of chicken nuggets offered)). | before and after intervention | Quantity of school meals consumed or wasted | Quantitative research articles evaluating interventions, initiatives, and policies to influence school meal consumption |
| 15 | Crockett 2018 ^28^ | Nutritional labelling for healthier food or non-alcoholic drink purchasing and consumption | To investigate whether nutritional labels (i.e., labels providing information about nutritional content) persuade people to buy or consume different (healthy) kinds of food | All age groups | 1.Nutritional labelling with energy (Calorie) information | No Intervention | Food purchasing and consumption | 1.Randomized controlled trial 2. Quasi experimental study 3.ITS |
| 16 | Daley 2020 ^43^ | Effects of physical activity calorie equivalent food labelling to reduce food selection and consumption: systematic review and meta-analysis of randomised controlled studies | Systematically search for randomised controlled trials and experimental studies of the effects of PACE food labelling on the selection, purchase or consumption of food/drinks. | NR | 1.Calorie labelling (Physical activity reduce Calorie level) 2. Portion size control (PACE labelling resulted in the public consuming less grams of food) | PACE labelling resulted in the public consuming less grams of food | NR | Randomized controlled trial |
| 17 | Ding 2020 ^44^ | Systematic Review on International Salt Reduction Policy in Restaurants | To review the restaurant salt reduction policies administered at the national or regional level around the world in order to provide suggestions for effective salt reduction in the catering and restaurant sectors. | NR | Labelling nutrients including sodium of menu items (Using icons including obvious graphic salt warnings to intuitively display whether the dish is good or bad for health) | NR | NR | Policies, programs, and initiatives issued by the government at all levels (of country and region) regarding reducing salt consumption from restaurants. The original policy documents published on the official sites and relevant news reports. Materials only proposing or negotiating salt reduction without specific strategies were excluded. |
| 18 | Driessen 2014 ^45^ | The effect of changes to the school food environment on eating behaviours and/or body weight in children: a systematic review | To systematically review the evidence relating to interventions that change the school food environment, with outcomes including both food-related behaviours (purchasing, consumption) and body weight. | Children/ Adolescents | 1. Reducing portion size (Reducing portion sizes and fat content of high fat/sugar-foods and beverages from all school food sources, reduced frequency of serving deep fried chips. Intervention to reduce sizes of sweetened beverages and chips and increasing the availability of water and reduced fat/baked chips. Nutrition and portion size standards for beverages and foods. | Comparator group with before and after | Change in weight or other | 1.Quasi experimental study 2. Cohort study |
| 19 | Espino 2015 ^46^ | Community-Based Restaurant Interventions to Promote Healthy Eating: A Systematic Review | To summarize and evaluates the evidence supporting community-based restaurant interventions | NR | Created and labelled menu; Labelled “healthy dining” menu items on the basis of program and Food and Drug Administration criteria | NR | NR | 1. Cohort study 2. Quasi experimental study 3. Cross sectional study |
| 20 | Fernandes 2016 ^47^ | Influence of menu labeling on food choices in real-life settings: a systematic review | To assess the influence of diverse menu-labelling formats on food choices in real-life settings | NR | Quantitative menu labelling (e.g., number of calories, nutrient content, proportion of calories from fat) or qualitative menu labelling (e.g., traffic-light labelling, healthy-food symbols, or messages) of meals and/or drinks, visibly displayed at points of selection (e.g., on menus, table displays, or menu boards, or beside food items at buffets, and counters) | No Intervention | Absolute number or proportion (%) of sales, purchases, choice of targeted items, food items/food groups, or different portion sizes purchased or selected on site | 1.Randomized controlled trial 2. Quasi experimental study 3. Cross sectional study |
| 21 | Funderburk 2020 ^24^ | Healthy Behaviors through Behavioral Design–Obesity Prevention | To explore the connections between the built environment and behaviour modification leading to healthy outcomes for the individual. | NR | 1.Food labelling that provided information on energy density and macronutrient content, point-of-purchasing labelling (1) (simple color-coded (red, amber, green) labelling intervention of food and beverages, meant to increase nutrition knowledge. The calorie and fat content of each portion of a food or beverage was used to code the items. Red coded foods were those that should be consumed less often; those that were amber consumed in moderation; and those that were green consumed frequently) | No intervention, other intervention, before/ after | Change in behaviours leading to healthy choices for nutrition and physical activity is a combination of the design of the environment and intentional programming | NR |
| 22 | Gittelsohn 2013 ^48^ | Community- based Interventions in Prepared- food sources: A Systematic Review | To systematically review community-based interventions in prepared food sources that aimed to increase access to and consumption of healthful foods. | All age groups | 1.Menu labelling; point-of-purchase materials (logo and signs, menu inserts) 2.Half-sized portions of entrées; fruit and vegetable side dishes; low-fat milk or water | NR | Prepared-food source impact results, Consumer impact measures (psychosocial, behavioural, and health outcomes), Process evaluation measures (dose, reach, and fidelity, which indicate how well the program was implemented according to plan), Feasibility assessment measures (acceptability, operability, and perceived sustainability) | NR |
| 23 | Gordon 2018 ^49^ | Healthier Choices in School Cafeterias: A Systematic Review of Cafeteria Interventions | To describe school cafeteria interventions in terms of a behavioural economics scheme and to assess which system is more likely to be effective in improving food selection or consumption. | Children | Calorie labelling (Emoticons, cartoon stickers, kiosk rating), Portion size control (Difference size dishware, increased portion size of fruits and vegetables) | NR | Body mass index (BMI) status or leads to a change of at least 30% in food- related behaviour | Randomized controlled trial |
| 24 | Grech 2015 ^50^ | A systematic literature review of nutrition interventions in vending machines that encourage consumers to make healthier choices | To determine the efficacy of nutrition interventions in vending machine in prompting dietary behaviour change to improve diet quality or weight status of the consumers of vended snacks compared with customers of vending machines where there is no change to the products typically sold. | NR | 1.Menu labelling (POP nutrition information: traffic light sticker with interpretive poster: green stickers: ‘Go ahead, eat and enjoy’. Yellow stickers: ‘Caution, eat moderately’, and red colour indicated ‘Stop, eat sparingly) | Other intervention/No intervention/ before or after | Dietary behaviour change | 1. Randomized controlled Trial 2. Quasi experimental study |
| 25 | Harbers 2020 (protocol by Beulens 2020) ^51^ | The effects of nudges on purchases, food choice, and energy intake or content of purchases in real-life food purchasing environments: a systematic review and evidence synthesis | Interventions in proximal physical micro-environments typology (TIPPME) to promote healthy purchases, food choice, or affecting energy intake or content of purchases, within real-life food purchasing environments. Second, we aimed to investigate the potentially moderating role of SEP. | Adults | 1.Calorie label, Nutrition label on menu boards 2. Portion size (Baseline 100% portion size, Intervention 150% portion size, availability of different sizes of sausages) | before or after | NR | 1.Randomized controlled trial 2. Quasi experimental study |
| 26 | Harnack 2008 ^52^ | Effect of point-of-purchase calorie labeling on restaurant and cafeteria food choices: A review of the literature | To review published research on the effect of point-of-purchase calorie labelling on cafeteria and restaurant menu food choices." | All age groups | 1.Calorie la belling | Other intervention/No intervention/ before or after | Food choices | 1. Cross sectional study 2. Quasi experimental study |
| 27 | Hendren 2017 ^25^ | Impact of worksite cafeteria interventions on fruit and vegetable consumption in adults A systematic review | To determine the effect of worksite cafeteria interventions on F/V consumption, with a secondary objective to identify interventions that result in long-term behaviour change. | Adults | 1.Point-of-purchase labelling (Nutrition display/ rotating calorie labelling, Traffic light labelling, Logos) 2. Portion size control labelling | Other intervention/No intervention/ before or after | Change in F/V consumption in worksite dining locations, long-term success in behaviour change. | 1. Randomized controlled Trial 2. Quasi experimental study 3. Randomized pre-/post-test experimental design 4. Unblinded controlled intervention |
| 28 | Hillier-Brown 2017 ^53^ | The impact of interventions to promote healthier ready-to-eat meals (to eat in, to take away or to be delivered) sold by specific food outlets open to the general public: a systematic review | To systematically review the impact of interventions to promote healthier ready to-eat meals (to eat in, to take away or to be delivered) sold by specific food outlets accessible to the general public. | NR | 1.calorie labelling (Signposting: Interventions that highlighted to customers the healthier, or less healthy, menu options available) 2. Portion Size (Changing pre-packed children’s meal content: Prepacked meal content changed to include healthier options, smaller portion sizes of less healthy options and/or removal of other less healthy options) | No intervention, before/ after | Dietary outcomes (e.g., energy intake), purchasing behaviour (e.g., sales data) and attitudes towards healthier menu choice and preferences. | 1.Randomized controlled trial 2. Quasi experimental study 3. Cohort study 4. Cross sectional study |
| 29 | Hollands 2015 ^54^ | Portion, package or tableware size for changing selection and consumption of food, alcohol and tobacco | Effects of interventions involving exposure to different sizes or sets of physical dimensions of a portion, package, individual unit or item of tableware on unregulated selection or consumption of food, alcohol or tobacco products in adults and children and to assess the extent to which these effects may be modified by study, intervention and participant characteristics." | All age groups | 1.Portion size (food portion size, tableware size) | Other intervention/No intervention | 1. Consumption (intake) of a product [amount of energy (e.g., calories), substances (e.g., carbon monoxide, alcohol, saturated fat), or products (e.g., food, drink or tobacco) consumed, measured in applicable natural units (e.g., kcals, kilojoules, grams). | Randomized controlled trial |
| 30 | Jaime 2009 ^55^ | Do school based food and nutrition policies improve diet and reduce obesity? | Effectiveness of school food and nutrition policies worldwide in improving the school food environment, student's dietary intake, and decreasing overweight and obesity. | All age groups | 1.Portion size (regulation on type and portion size of foods vending in school) | NR | menu composition, availability and sales of food and beverages at school, student's dietary intake and BMI. | Quasi experimental study |
| 31 | Kraak 2019 ^56^ | Progress Evaluation for Transnational Restaurant Chains to Reformulate Products and Standardize Portions to Meet Healthy Dietary Guidelines and Reduce Obesity and Non-Communicable Disease Risks, 2000–2018: A Scoping and Systematic Review to Inform Policy | To describe the transnational restaurant industry structure and eating trends, summarize results from a scoping review of healthy dietary guidelines for restaurants and a systematic review of five electronic databases (2000–2018) to identify studies on nutrient profile and portion size changes made by transnational restaurants over 18 years. | NR | 1.Energy (calories or kilojoules or energy density); Fats (total, saturated, and TFA); Sugars (total, added, or free); and Sodium (salt or sodium density) 2. Portion Size (Meals, non-alcoholic beverages or drinks, side dishes, desserts, or other edible products.) | Other intervention | Nutrient profile or composition: Energy; Fats; Sugars; and Sodium. Portion or serving size: Meals | Longitudinal, cross-sectional, descriptive, observational, and/or intervention studies. |
| 32 | Littlewood 2016 ^57^ | Menu labelling is effective in reducing energy ordered and consumed: a systematic review and meta-analysis of recent studies | The effect of menu labelling regarding changes in energy consumed, ordered or selected in both real-world and experimental settings. | All age groups | 1.Energy labelling | Other intervention/No intervention/ before or after | energy consumed, ordered or selected, as quantified kJ/kcal differences. | 1.Randomized controlled trial 2. Quasi experimental study 3. Cross sectional study |
| 33 | Long 2015 ^58^ | Systematic Review and Meta-analysis of the Impact of Restaurant Menu Calorie Labeling | Impact of menu calorie labelling with or without a daily anchor statement compared with menus without calorie labelling on calories ordered, purchased, or consumed during the meal as well as impact on total daily energy intake or weight or body mass index | All age groups | 1.Calorie labelling | Comparator group with No intervention/ before or after | Change in the number of calories in a single meal ordered or purchased with and without menu calorie labelling. | 1. Randomized controlled Trial 2. Quasi experimental study |
| 34 | Lycett 2017 ^59^ | ‘Nudge’ interventions for improving children's dietary behaviors in the home: A systematic review | To systematically review the effectiveness of nudge interventions designed to improve children's dietary behaviours within the family home (or another environment if judged transferable to the home). | Children | 1.Nutritional information in monochrome or in traffic light colours | Other or no intervention | Improvements in diet related behaviour- change on preference, purchase, selection or consumption of healthy foods | 1.Randomized controlled trial 2. Quasi experimental study |
| 35 | Mandracchia 2021 ^60^ | Interventions to Promote Healthy Meals in Full-Service Restaurants and Canteens: A Systematic Review and Meta-Analysis | The effectiveness of full-service restaurant and canteen-based interventions in increasing the dietary intake, food availability, and food purchase of healthy meals. | All age groups | 1.Menu Labelling (menu board plus poster labelling) | NR | Increasing the offerings and demand for healthier meals as the primary or secondary outcome | Randomized controlled trial |
| 36 | Marcano-Olivier 2020 ^61^ | Using Nudges to Promote Healthy Food Choices in the School Dining Room: A Systematic Review of Previous Investigations | A systematic review of studies that used behavioural nudges to promote a healthy school cafeteria environment. | All age groups | 1.Portion size (weights used to approximate consumption, Fruit, vegetable selection and consumption of whole portion) | NR | Reported outcome measure for food selection or consumption, | Cohort study |
| 37 | Melian-Fleitas 2021 ^62^ | Influence of Nutrition, Food and Diet-Related Interventions in the Workplace: A Meta-Analysis with Meta-Regression | To review the scientific literature on the influence of verified nutrition, food and diet interventions on occupational health. | Adults | 1.Menu Labelling 2. Traffic lighting | NR | NR | Cross-sectional descriptive study and critical analysis |
| 38 | Metcalfe 2020 ^63^ | A systematic review of school meal nudge interventions to improve youth food behaviors | To conduct a systematic review to determine the range and quality of available evidence of school meal nudges on student eating behaviours, such as school meal participation, food selection, consumption, and waste. | Children | 1.calorie labelling at point of purchase 2. Portion size (increased portion sizes of fruits and vegetables) | 1. Took more calorie in comparator 2. Increase selection of healthy foods from pre to post intervention | Selection, consumption, waste, or school meal participation. | 1.Non-Randomized controlled Trial 2. Non controlled Trial 3. Cross over study. |
| 39 | Micha 2018 ^64^ | Effectiveness of school food environment policies on children’s dietary behaviors: A systematic review and meta-analysis | To systematically review and quantify the impact of school food environment policies on dietary habits, adiposity, and metabolic risk in children. | Children | 1.Calorie labelling | NR | food/beverage standards reduced sugar- sweetened beverage intake and unhealthy snacks without effects on total calories. School meal standards (mainly lunch) increased fruit intake and reduced total fat, saturated fat and sodium but not total calories) | Quasi experimental study |
| 40 | Mingay 2022 ^65^ | The Impact of Modifying Food Service Practices in Secondary Schools Providing a Routine Meal Service on Student’s Food Behaviours, Health and Dining Experience: A Systematic Review and Meta-Analysis | To examine nutrition interventions within secondary school dining rooms that provide a routine meal service; the intervention strategies implemented and outcomes measured, and the impact on adolescents’ food behaviours, health and dining experience specifically within this setting | Adolescent | 1.Nutritional information / calorie labels (POS signage with nutritional information / calorie labels; Installation of kiosk stations with Nutri-Advice software for children to assess and select a well-balanced meal from daily food available on cafeteria menu) 2. Portion size (restrict portion size of high-fat and sugar snacks, SSBs and the fat content of all foods served; limit frequency of serving high-fat veg) | No intervention, before/ after | Selection or consumption of a meal component (a food item, food group or nutrient), qualitative feedback, attitudes or satisfaction scores, knowledge, school meal program participation rates. | 1. Randomized controlled Trial 2. Quasi experimental study |
| 41 | Moran 2020 ^66^ | Associations between Governmental Policies to Improve the Nutritional Quality of Supermarket Purchases and Individual, Retailer, and Community Health Outcomes: An Integrative Review | To identify governmental policies in the United States (U.S.), designed to promote healthy choices in supermarkets; and (2) synthesize evidence of these policies’ effects on retailers, consumers, and community health. | All age groups | 1.Calorie labelling | NR | NR | 1.Quasi experimental study 2. Descriptive study |
| 42 | Naicker 2021 ^67^ | Workplace cafeteria and other multicomponent interventions to promote healthy eating among adults: A systematic review | To identify and assess the effectiveness of workplace cafeteria and other supporting multicomponent interventions to promote healthy eating. | Adults | 1.Nutrition labelling 2. Traffic lighting keyhole lighting 3. Portion size (Changes included reducing the size of serving spoons (BL) and offering half portions at half price.) | Other intervention/No intervention | Food quality or quantity, targeting client’s information, education or motivation and targeting food choice at point of purchase. | 1.Randomized controlled trial 2. Quasi Experimental study 3. Non randomized trial 4. Time series 5. Pre post |
| 43 | Nikolau 2015 ^68^ | Calorie-labelling: Does it impact on calorie purchase in catering outlets and the views of young adults? | To review the current literature, conduct a meta-analysis and determine young adults’ views on calorie-labelling and on calories purchased. | Adults | 1.Calorie labelling | NR | Calories purchased | 1. Cross sectional study 2. Quasi experimental study 3. Natural experimental |
| 44 | Osei-Assibey 2012 ^69^ | The influence of the food environment on overweight and obesity in young children: a systematic review | To examine the evidence for environmental influences on dietary determinants of obesity, focusing on younger children (birth to 8 years). | Children | 1.Portion size (Doubling the portion size of the main dish served at lunchtime increased the food consumed by 25% and energy intake by 15%.) | NR | BMI/weight, skin-fold thickness, percentage body fat, per cent overweight/obesity or dietary behaviours linked to obesity. | 1. Crossover study 2. Non RCT |
| 45 | Park 2021 ^70^ | Tableware and Food Consumption | To investigate whether the evidence for an association between tableware in the built food environment and food consumption is consistent and important. | All age groups | 1.Portion size (the shape and size of tableware can influence how much is served, the size of food portions served to construct a platescape is related to the amount of food actually consumed. The shape and size of plates or bowls delineate norms for appropriate amounts of food to eat at a meal. Finally, the shape and size of glasses or cups also influence the amount of liquid people perceive) | NR | Food consumption | Randomized controlled trial |
| 46 | Pineda 2021 ^71^ | Improving the school food environment for the prevention of childhood obesity: What works and what doesn't | 1.We have designed a new systematic review, meta-analysis, and meta-regression aimed at assessing the effectiveness of school food environment interventions in the prevention of childhood obesity, with a focus on adiposity and dietary intake outcomes, based on studies published up to 2020. 2. Effective school food environment interventions for the prevention of childhood obesity: systematic review and meta-analysis | Children | Calorie labelling | NR | Weight and height, fat mass percent, BMI, ponderal index, skin-fold thickness, waist circumference, waist–hip ratio, prevalence of overweight and obesity, BMI z score, dietary intake, and food purchasing. | Quasi experimental study |
| 47 | Querstret 2016 ^72^ | Environmental interventions for altering eating behaviours of employees in the workplace: a systematic review | To evaluate and synthesize the evidence surrounding the effectiveness of environmental interventions targeting eating behaviour in the workplace | All age groups | 1.Labelling of healthy food and calorie labelling 2. Healthy changes to content and size of food options | Before or after | Behavioural measures of eating behaviour or physiological measures associated with eating behaviour. | 1. Randomized controlled Trial 2. Quasi experimental study |
| 48 | Rachmah 2022 ^73^ | The effectiveness of nutrition and health intervention in workplace setting: A systematic review | to review the efficacy of health and nutrition intervention among workers that result can be used as the evidence based for policymakers to start implementing health and nutrition education periodically. | Adults | 1. Environmental Modification including Portion size control | NR | Intakes of saturated fat, salt and nutrition knowledge, changes in BMI | Cluster controlled study |
| 49 | Reynolds 2022 (Protocol by the same name) ^74^ ^23^ | Communicating evidence about the environment’s role in obesity and support for government policies to tackle obesity: a systematic review with meta-analysis | To investigate whether communicating information about environmental factors that influence obesity changes support for polices that aim to reduce obesity. | NR | 1.calorie label in restaurants menu 2. Portion size (changing the availability of larger portion sizes) | Other intervention/ No intervention | Beliefs about the causes of obesity including: (a) the belief that the obesogenic environment/society/food industry is responsible for obesity; (b) the belief that willpower/self-control/personal responsibility is responsible for obesity; and (c) the belief that genetics/biology/heredity is responsible for obesity | NR |
| 50 | Richardson 2022 ^75^ | Availability of Healthy Food and Beverages in Hospital Outlets and Interventions in the UK and USA to Improve the Hospital Food Environment: A Systematic Narrative Literature Review | The extent to which healthy food and drink options are available to employees and visitors in hospital food environments and to determine which interventions are effective in reducing the purchase and consumption of unhealthy foods and beverages. | NR | 1.Nutrition labelling 2. Traffic light 3. Portion Size control | No intervention/ before or after | Nutritional quality of food and beverages available to employees and visitors in hospitals | 1. Randomized controlled Trial 2. Quasi experimental study 3. Cohort study |
| 51 | Rosenheck 2008 ^76^ | Fast food consumption and increased caloric intake: a systematic review of a trajectory towards weight gain and obesity risk | to examine the existing evidence investigating an association between fast food consumption and increased caloric intake leading to weight gain and obesity to facilitate future research and public health recommendations. | All age groups | 1.Portion Size (Serving size (one large vs 4 small at 15 mins interval vs 4 small at once)) | NR | Changes in caloric intake | Quasi experimental study |
| 52 | Roy 2015 ^77^ | Food Environment Interventions to Improve the Dietary Behavior of Young Adults in Tertiary Education Settings: A Systematic Literature Review | the effective intervention strategies that have been conducted to improve the dietary behaviour of young adults through food environment changes in university/college settings. | Adults | 1.Nutrition Labelling 2. Star rating, Traffic light 3. Portion Size control (Std pack vs 100kcal pack, decreasing weight from 88gm over 3 wks, increasing FV intake) | No intervention/ before or after | Changes to healthier food choices, reductions in unhealthy food choices, nutrition knowledge, and/or food/drink sales; | 1.Randomized controlled trial 2. Quasi experimental study 3. Cross sectional study |
| 53 | Sacco 2017 ^78^ | The influence of menu labelling on food choices among children and adolescents: a systematic review of the literature | to assess whether menu labelling influences the amount of calories ordered by children and adolescents (or parents on behalf of youth) in food outlets including restaurants and cafeterias. | All age groups | 1. Menu labelling (Calorie content, Nutrition information) 2. Heart/apple symbol indicating healthy choice, Nutrition bargain price, traffic light 3. Average daily caloric requirements for adults | No intervention/ before or after | Changes in calorie intake | 1.Randomized controlled trial 2. Quasi experimental study 3.cross sectional study |
| 54 | Sarink 2016 ^79^ | The impact of menu energy labelling across socioeconomic groups: A systematic review | to review evidence of the impact of menu energy labelling across socioeconomic strata. | Adults | 1.Calorie labelling | Comparator group before or after | Choice, purchase of calories | Quasi experimental study |
| 55 | Sawada 2019 ^80^ | Social marketing including financial incentive programs at worksite cafeterias for preventing obesity: a systematic review | The effect of health outcomes or food intake behaviour at the population level of financial incentive policies applied to workplace cafeterias, vending machines, or kiosks in preventing obesity among employees. | Adults | 1.Menu Labelling (1.A smaller portion size was added to the assortment and value-size pricing (a lower price per unit for large portions than for small portions).2. Labelled as low or very low in energy density)3. Explanation of traffic light system or the proportion of employee’s traffic light group purchases) 4. Portion size | NR | Changes in weight (kg), body mass index (BMI) (kg/m2), and changes in hemoglobin A1c (HbA1c) (%). The secondary outcomes were blood pressure (mmHg), changes in cholesterol levels (mg), food consumption (changes in vegetable consumption [g or serving (SV)], changes in fruit consumption [g or SV], changes in fruit and vegetable consumption [g or SV], changes in the consumption of sugary beverages[g], changes in the consumption of sweets [g] and other foods [g] ), and nutritional intake (changes in fat and oil intake [g], changes in fibre intake [g], and changes in energy intake [kcal]). | 1.Randomized controlled trial 2. Cluster randomized controlled trial |
| 56 | Schuez 2021 ^81^ | Equity effects of dietary nudging field experiments | To provide such equity analyses of existing real-world interventions that employ nudging strategies to change dietary behaviours. | NR | 1.Nutrition labelling 2. Portion size control (Affectively oriented: Healthy Eating Call) | No intervention/ before or after | Number of healthy food orders | NR |
| 57 | Seyedhamzeh 2018 ^82^ | Physical activity equivalent labeling vs. calorie labeling: a systematic review and meta-analysis | To identify and evaluate the published literature comparing effects on health behaviour between physical activity equivalent labelling and calorie-only labelling | Adults | 1.Calorie labelling (Physical activity equivalent) | Comparator group with no intervention (Physical activity equivalent/calorie labelling) | Calorie ordered | Quasi experimental study |
| 58 | Seymour 2004 ^83^ | Impact of nutrition environmental interventions on point-of-purchase behavior in adults: a review | To identify the strengths and weaknesses of the research related to environmental and policy nutrition interventions, summarize the findings of this research, and identify areas for future research. | All age groups | 1.Calorie labelling 2. Portion size (decreasing portion) | No intervention/ before or after | Change in behaviour through sales data, dietary assessment, or physiologic changes | Quasi experimental study |
| 59 | Shepherd 2006 ^84^ | Young people and healthy eating: a systematic review of research on barriers and facilitators | To examine the barriers to, and facilitators of, healthy eating among young people (11–16 years) | All age groups | Nutritional labelling | No Intervention | Increased food consumption | 1.Randomized controlled trial 2. Quasi experimental study |
| 60 | Sinclair 2014 ^85^ | The Influence of Menu Labeling on Calories Selected or Consumed: A Systematic Review and Meta-Analysis | To determine whether or not the current evidence, when limited to studies with a control or comparison group, supports menu-based nutrition information for the selection or consumption of fewer calories ii. to determine if the format of the nutrition information (informative vs contextual/interpretive) and influenced calories selected or consumed" and influenced calories selected or consumed | All age groups | 1.Calorie labelling 2. Heart symbol next to three low-fat items, exercise equivalents to the caloric content label, traffic light 3. Recommended daily caloric intakes | Comparator group with No intervention/ before or after | Consumers’ response to menu labelling | Quasi experimental study |
| 61 | Sisnowski 2017 (Protocol by the same name) ^86^ ^86^ | Improving food environments and tackling obesity: A realist systematic review of the policy success of regulatory interventions targeting population nutrition | To investigate the effect of real-world policies targeting different aspects of the food environment that shape individual and collective nutrition. | All age groups | 1.Calorie labelling | No intervention/ before or after | the effect of statutory provisions reducing the consumption of energy-dense foods and beverages | 1.Cohort study 2. Cross sectional study 3. Quasi experimental study |
| 62 | Stiles 2022 ^87^ | Effectiveness of Strategies to Decrease Animal-Sourced Protein and/or Increase Plant-Sourced Protein in Foodservice Settings: A Systematic Literature Review | Effective strategies to decrease animal protein and/or increase plant protein in foodservice settings on uptake, satisfaction, financial, environmental, and dietary intake outcomes. | All age groups | 1.Tastefocused (mouth-watering grilled vegetable wrap) and health focused labelling (healthy choice vegetable wrap) 2. Portion size control (reducing the meat/fish Portion and increasing the vegetables portion) | Other intervention/ No intervention | Consumer acceptance of the intervention (e.g., meal purchases, plate waste, reported uptake), satisfaction, financial and environmental measures | 1.Randomized controlled trial 2. Mixed method study |
| 63 | Swartz 2011 ^88^ | Calorie menu labeling on quick-service restaurant menus: an updated systematic review of the literature | The purpose of this paper is to use current literature to answer the question of whether calorie labelling on menus at restaurants and cafeterias has an effect on consumer purchasing and eating behaviours | All age groups | 1.Calorie labelling | Other intervention/ No intervention/ before or after | Calorie ordering and purchasing | 1. Randomized controlled Trial 2. Quasi experimental study 3. Cross sectional study 4. Natural experiment, pre/ post intervention |
| 64 | Thorpe 2021 ^89^ | Business outcomes of healthy food service initiatives in schools: A systematic review | The aims of this systematic review were to (i) synthesize the evidence to date on the effect of school food service initiatives on business outcomes, including commercial viability, stakeholder perceptions and customer perceptions, and initiative maintenance and scale-up; (ii) examine whether business outcomes vary by the type or duration of initiative; and (iii) explore the relationship between business outcomes and health behaviour outcomes of healthy food retail initiative in schools | NR | Menu labelling encourage the healthier choices | No Intervention | Commercial viability/ stakeholder perceptions/ customer perceptions/initiative maintenance and scale-up | Randomized controlled trial |
| 65 | Tran 2021 (Protocol by Ananthapavan 2021) ^90^ | A Systematic Review of Economic Evaluations of Health-Promoting Food Retail-Based Interventions | The aim of this systematic review is to: (i) assess the evidence of cost-effectiveness of food retail interventions to improve diet-related health outcomes; and (ii) identify the key assumptions used to conduct economic evaluations of food retail-based interventions. | All age groups | 1.Calorie labelling (caloric value of food translated to number of minutes to perform occupational activity) | Other intervention/ No intervention | Diet or health related, cost per healthy food item purchased/ served or cost per disability-adjusted life year (DALY) averted. Cost-effectiveness analysis (CEA), cost-utility analysis (CUA), cost-benefit analysis, and cost minimisation analysis); Modelled DALYs, Modelled DALYs averted, DALYs averted, Modelled BMI, Modelled deaths from coronary heart disease prevented or postponed | Modelled policy interventions (individual-level microsimulation model, multi-state Markov modelling over a lifetime time horizon) |
| 66 | Von Philipsborn 2019 ^29^ | Environmental interventions to reduce the consumption of sugar sweetened beverages and their effects on health | The effects of environmental interventions (excluding taxation) on the consumption of sugar-sweetened beverages and sugar-sweetened milk, diet-related anthropometric measures and health outcomes, and on any reported unintended consequences or adverse outcomes | All age groups | 1.Calorie labelling 2. Traffic light | NR | Direct and indirect measures of SSB intake, Diet-related anthropometric measures and health outcomes Secondary outcome: Measures of financial and economic viability, Diet-related psychosocial variables, Target group perceptions of the intervention, Consumption of beverages other than SSB | RTCs, NRCTs, CBA, ITS, RMS |
| 67 | Whatnall 2020 (Protocol by Hutchesson 2020) ^91^ | Effectiveness of Nutrition Interventions in Vending Machines to Encourage the Purchase and Consumption of Healthier Food and Drinks in the University Setting: A Systematic Review | To evaluate the current evidence examining the effectiveness of nutrition interventions in vending machines to encourage the purchase and consumption of healthier food and drinks in the university setting. | NR | 1.Menu Labelling (Nutritional information) 2. Color-coded stickers placed on items (red, yellow, green) to indicate health rating, with a larger sticker on the front of machines explaining the color-coding and matching nutrition information. | Other intervention/ No intervention | Purchase/sales of food and drinks from vending machines, dietary behaviour change, change in food/drinks available within vending machines | Randomized controlled Trial 2. Quasi experimental study |
| 68 | Wilson 2016 ^92^ | Nudging healthier food and beverage choices through salience and priming. Evidence from a systematic review | The effectiveness of nudging for influencing food and beverage choices and to determine the extent to which nudging terminology was used in the literature | Adults | 1.Calorie labelling 2. Traffic light 3. Portion size (altered serving utensils) | Other intervention/ No intervention | Outcome of interest are food and beverage choices | 1.Randomized controlled trial 2. Cross-sectional 3. Case control |
| 69 | Wyse 2021 ^30^ | The Effectiveness of Interventions Delivered Using Digital Food Environments to Encourage Healthy Food Choices: A Systematic Review and Meta-Analysis | The impact of dietary interventions embedded within online food ordering systems on user purchasing of healthier foods and beverages and to identify any unintended adverse consequences and describe the cost and cost effectiveness of the included interventions. | All age groups | 1.Menu Labelling (Nutrient content (Calories, fat) 2. Traffic light labels, Nutri--score labels) 3. Statements related to meal components selected such as "Your meal does not look like a balanced meal" if a food group e.g., fruit, vegetables was not selected. | No intervention (i.e., true control), a delayed intervention (i.e., wait-list control), usual care, or an alternative intervention that did not seek to influence food purchasing behaviour, and/or was not delivered using an online food ordering system | " Food and beverage purchases according to food groups, food categories or target items (e.g. sugar sweetened beverages); Macro- and micronutrient content of food/beverage purchases (e.g. mean energy, saturated fat, total sugar or sodium; or % energy contributed from fat or sugar; or energy density; Macro- and micronutrient content of food/beverage purchases (e.g. mean energy, saturated fat, total sugar or sodium; or % energy contributed from fat or sugar; or energy density; | Randomized controlled trial |

## Table. S4: Characteristics of excluded studies - completed reviews (n=92)

| **S.No.** | **Study ID** | **Title** | **Reason for exclusion** |
| --- | --- | --- | --- |
| 1 | Andueza 2022 | Effectiveness of Nutritional Strategies on Improving the Quality of Diet of Children from 6 to 12 Years Old: A Systematic Review | Wrong intervention |
| 2 | Li 2022 | How Does the University Food Environment Impact Student Dietary Behaviors? A Systematic Review | Wrong intervention |
| 3 | Liu 2020 | Health and Economic Impacts of the National Menu Calorie Labeling Law in the United States: A Microsimulation Study | Wrong study design |
| 4 | Slapø 2021 | Efficiency of In-Store Interventions to Impact Customers to Purchase Healthier Food and Beverage Products in Real-Life Grocery Stores: A Systematic Review and Meta-Analysis. | Wrong intervention |
| 5 | Abbott 2013 | Effectiveness of mealtime interventions on nutritional outcomes for the elderly living in residential care: A systematic review and meta-analysis | Wrong intervention |
| 6 | Adam 2016 | What is the effectiveness of obesity related interventions at retail grocery stores and supermarkets? -a systematic review | Wrong intervention |
| 7 | Anderson 2009 | The effectiveness of worksite nutrition and physical activity interventions for controlling employee overweight and obesity: a systematic review. | Wrong intervention (mostly counselling no menu labelling or portion size) |
| 8 | Anderson 2021 | Improving Healthy Food Choices in Low-Income Settings in the United States Using Behavioral Economic-Based Adaptations to Choice Architecture. | Wrong study design |
| 9 | Barnes 2021 | Improving implementation of school-based healthy eating and physical activity policies, practices, and programs: a systematic review. | Wrong intervention |
| 10 | Bianchi 2018 | Effectiveness of interventions restructuring the physical environment to reduce the consumption, purchase, or selection of meat products: protocol for a systematic review with narrative synthesis | Wrong intervention |
| 11 | Bivoltsis 2018 | Food environments and dietary intakes among adults: does the type of spatial exposure measurement matter? A systematic review | Wrong intervention (wrong exposure as the reason for exclusion spatial study) |
| 12 | Blanchette 2005 | Determinants of fruit and vegetable consumption among 6-12-year-old children and effective interventions to increase consumption | Wrong intervention |
| 13 | Browne 2020 | Effects of food policy actions on Indigenous Peoples’ nutrition-related outcomes: a systematic review | Wrong intervention |
| 14 | Cardozo 2022 | Food environment and excess weight in schoolchildren: a South American systematic review | Wrong intervention |
| 15 | Carducci 2017 | Food environments of school-aged children and adolescents in low-and middle-income countries: Investigating a proposed conceptual framework | Wrong intervention |
| 16 | Carter 2016 | Provision of information-based cues at point-of-choice for food, alcohol and tobacco selection and consumption: protocol for a systematic review | Wrong intervention |
| 17 | Chavez-Ugalde 2021 | Conceptualizing the commercial determinants of dietary behaviors associated with obesity: A systematic review using principles from critical interpretative synthesis | Wrong intervention |
| 18 | Christoforou 2013 | Effectiveness of community interventions for the reduction of dietary salt intake: A systematic review | Wrong intervention |
| 19 | Christoph 2016 | Correlates of nutrition label use among college students and young adults: a review | Wrong intervention |
| 20 | Da-Costa-Peres 2020 | Retail food environment around schools and overweight: A systematic review | Wrong intervention |
| 21 | Dabravolskaj 2020 | Effectiveness of school-based health promotion interventions prioritized by stakeholders from health and education sectors: a systematic review and meta-analysis | Wrong intervention |
| 22 | deAlbuquerque 2022 | Retail food outlets and metabolic syndrome: a systematic review of longitudinal studies | Wrong intervention |
| 23 | DeSa 2008 | Will European agricultural policy for school fruit and vegetables improve public health? A review of school fruit and vegetable programmes | Wrong intervention |
| 24 | Doyle 2016 | Systematic review of oral nutrition interventions and their effect on nutritional outcomes and satisfaction of adult cancer patients | Wrong intervention |
| 25 | Engler-Stringer 2014 | The community and consumer food environment and children's diet: a systematic review | Wrong intervention |
| 26 | Fergus 2021 | Nutrition Interventions in Low-Income Rural and Urban Retail Environments: A Systematic Review. | Wrong intervention |
| 27 | French 2003 | Environmental interventions to promote vegetable and fruit consumption among youth in school settings | Wrong setting |
| 28 | Glanz 2004 | Increasing fruit and vegetable intake by changing environments, policy and pricing: restaurant-based research, strategies, and recommendations | Wrong study design |
| 29 | Gonçalves 2021 | The food environment in schools and their immediate vicinities associated with excess weight in adolescence: A systematic review and meta-analysis | Wrong intervention |
| 30 | Gortmaker 2015 | Three interventions that reduce childhood obesity are projected to save more than they cost to implement | Wrong study design |
| 31 | Greene 2022 | Nutrition Interventions Addressing Structural Racism: A Scoping Review. | Wrong study design |
| 32 | Gressier 2019 | What is the impact of food reformulation on individual’s behaviour, nutrient intakes and health status? A systematic review of empirical evidence | Wrong intervention |
| 33 | Grieger 2016 | Discrete strategies to reduce intake of discretionary food choices: a scoping review | Wrong study design |
| 34 | Guedes De Vasconcelos 2011 | A systematic review of school-based interventions for obesity reduction in children and adolescents | Wrong study design |
| 35 | Hempel 2019 | Obesity Prevention Interventions and Implications for Energy Balance in the United States and Mexico: A Systematic Review of the Evidence and Meta-Analysis | Wrong intervention |
| 36 | Hillier-Brown 2017 | A description of interventions promoting healthier ready-to-eat meals (to eat in, to take away, or to be delivered) sold by specific food outlets in England: a systematic mapping and evidence synthesis. | Wrong intervention |
| 37 | Hollands 2015 | Portion, package or tableware size for changing selection and consumption of food, alcohol and tobacco | Wrong intervention |
| 38 | Houghtaling 2021 | A rapid review of stocking and marketing practices used to sell sugar-sweetened beverages in U.S. food stores. | Wrong setting |
| 39 | Houghtaling 2021 | Current Practices in the Stocking and Marketing of Sugar-Sweetened Beverages and Opportunities for Favorable Food Retail Change to Improve Consumer Diet Quality: A Rapid Review | Duplicate of no. 38 |
| 40 | Hyseni 2015 | Effectiveness of policy actions to improve population dietary patterns and prevent diet related non-communicable diseases: scoping review | Wrong study design |
| 41 | Hyseni 2017 | The effects of policy actions to improve population dietary patterns and prevent diet-related non-communicable diseases: Scoping review | Wrong study design |
| 42 | Kerins 2020 | Barriers and facilitators to implementation of menu labelling interventions from a food service industry perspective: a mixed methods systematic review | Wrong patient population |
| 43 | Kerins 2020 | Barriers and facilitators to implementation of menu labelling interventions to support healthy food choices: a mixed methods systematic review protocol | Wrong patient population |
| 44 | Kerr 2019 | Nutrition-related interventions targeting childhood overweight and obesity: A narrative review. | Wrong study design |
| 45 | Klingberg 2019 | Systematic review of childhood obesity prevention interventions in African countries | Wrong intervention |
| 46 | Kraak 2017 | A novel marketing mix and choice architecture framework to nudge restaurant customers toward healthy food environments to reduce obesity in the United States | Wrong intervention |
| 47 | Lambert 2009 | A review of school nutrition interventions globally as an evidence base for the development of the HealthKick programme in the Western Cape, South Africa: original research | Wrong intervention |
| 48 | Langellier 2019 | Complex Systems Approaches to Diet: A Systematic Review | Wrong intervention |
| 49 | Lee 2021 | Toward a Healthy and Environmentally Sustainable Campus Food Environment: A Scoping Review of Postsecondary Food Interventions. | Wrong study design |
| 50 | Liberato 2014 | Nutrition interventions at point-of-sale to encourage healthier food purchasing: a systematic review. | Wrong intervention |
| 51 | Liu 2021 | The Effect of Downsizing Packages of Energy-Dense, Nutrient-Poor Snacks and Drinks on Consumption, Intentions, and Perceptions-A Scoping Review | Wrong study design |
| 52 | Mackenbach 2019 | The association between the food environment and dietary patterns in different socio-economic groups: an overview of observational studies | Wrong intervention |
| 53 | Mackenbach 2022 | Relation between the food environment and oral health-systematic review | Wrong intervention (Front of pack label only) |
| 54 | Mafioletti 2017 | Trans fatty acids: After 14 years of Brazilian legislation are the labeling adequated? | Wrong intervention |
| 55 | Mah 2019 | A Systematic Review of the Effect of Retail Food Environment Interventions on Diet and Health with a Focus on the Enabling Role of Public Policies | Wrong intervention |
| 56 | Martin 2018 | Physical activity, diet and other behavioural interventions for improving cognition and school achievement in children and adolescents with obesity or overweight. | Wrong intervention |
| 57 | Meiklejohn 2016 | A Systematic Review of the Impact of Multi-Strategy Nutrition Education Programs on Health and Nutrition of Adolescents | Wrong intervention |
| 58 | Melnyk 2009 | The latest evidence to guide obesity prevention, policy, and clinical practice with overweight children and adolescents | Wrong study design |
| 59 | Monroy-Parada 2021 | [Map of school nutritional policies in Spain]. | Wrong study design |
| 60 | Needham 2018 | The characteristics of Australian food environments, diet and obesity: a systematic review | Wrong intervention |
| 61 | Ng 2022 | Identifying barriers and facilitators in the development and implementation of government-led food environment policies: a systematic review | Wrong study design |
| 62 | Nguyen 2021 | Implementing Food Environment Policies at Scale: What Helps? What Hinders? A Systematic Review of Barriers and Enablers. | Wrong intervention |
| 63 | Olstad 2017 | Can policy ameliorate socioeconomic disparities in obesity and obesity-related behaviours? A systematic review of policy interventions in adults and children | Wrong intervention |
| 64 | Ottrey 2016 | Hospital menu interventions: a systematic review of research | Wrong intervention |
| 65 | Penney 2015 | Local food environment interventions to improve healthy food choice in adults: a systematic review and realist synthesis protocol. | Wrong intervention |
| 66 | Perez-Ferrer 2019 | The food environment in Latin America: a systematic review with a focus on environments relevant to obesity and related chronic diseases | Wrong intervention |
| 67 | Qingzhou Liu 2022 | Consumer perception of appropriateness of discretionary food portion sizes – a systematic review protocol | Wrong intervention |
| 68 | Rincón-Gallardo 2020 | Effects of Menu Labeling Policies on Transnational Restaurant Chains to Promote a Healthy Diet: A Scoping Review to Inform Policy and Research | Wrong study design |
| 69 | Rose 2019 | The impact of and views on school food policy in young people aged 11-18 years in Europe: a mixed methods systematic review | Wrong intervention |
| 70 | Roudsari 2019 | A review of evidence-informed policy making in sustainable healthy food and nutrition systems | Wrong intervention |
| 71 | Saleem 2022 | Non-communicable diseases, type 2 diabetes, and influence of front of package nutrition labels on consumer's behaviour: Reformulations and future scope. | Wrong study design |
| 72 | Samoggia 2021 | Innovative Digital Technologies for Purchasing and Consumption in Urban and Regional Agro-Food Systems: A Systematic Review. | Wrong intervention |
| 73 | Shen 2019 | [Food environment and its relation to diet behavior and obesity in China]. | Wrong intervention |
| 74 | Souza 2018 | Association between the food environment and cardiometabolic risk factors: a systematic review and meta-analysis | Wrong intervention |
| 75 | Stevenson 2019 | Neighbourhood retail food outlet access, diet and body mass index in Canada: a systematic review. | Wrong intervention |
| 76 | Steyn 2009 | A review of school nutrition interventions globally as an evidence base for the development of the HealthKick programme in the Western Cape, South Africa | Wrong intervention |
| 77 | Steyn 2009 | Nutrition interventions in the workplace: Evidence of best practice | Wrong intervention |
| 78 | Suthers 2018 | Key Characteristics of Public Health Interventions Aimed at Increasing Whole Grain Intake: A Systematic Review | Wrong intervention |
| 79 | Tørris 2019 | Improving cardiovascular health through nudging healthier food choices: A systematic review | Wrong study design |
| 80 | Trieu 2015 | Salt Reduction Initiatives around the World - A Systematic Review of Progress towards the Global Target. | Wrong intervention |
| 81 | Van Epps 2016 | Restaurant Menu Labeling Policy: Review of Evidence and Controversies | Wrong study design |
| 82 | Vandevijvere 2019 | Effect of Formulation, Labelling, and Taxation Policies on the Nutritional Quality of the Food Supply | Wrong study design |
| 83 | Vargas 2022 | Co-created strategies that aim to improve the healthiness of food retail outlets internationally: a systematic narrative synthesis review | Wrong intervention |
| 84 | Verrotti 2014 | Childhood obesity: prevention and strategies of intervention. A systematic review of school-based interventions in primary schools. | Wrong intervention |
| 85 | Vivian S.S. 2021 | Association between school food environment and overweight in adolescence: a systematic review | Wrong intervention |
| 86 | Von Philipsborn 2019 | Environmental interventions to reduce the consumption of sugar-sweetened beverages and their effects on health | Duplicate record |
| 87 | Waters 2011 | Interventions for preventing obesity in children | Wrong intervention |
| 88 | Westbury 2021 | The influence of the urban food environment on diet, nutrition, and health outcomes in low and middle-income countries: A systematic review | Wrong intervention |
| 89 | Wolfenden 2018 #288 / Protocol #575 | Strategies to improve the implementation of workplace-based policies or practices targeting tobacco, alcohol, diet, physical activity and obesity. | Wrong intervention |
| 90 | Wolfenden 2020 #1400 / duplicate #592/ protocol #287 | Strategies to improve the implementation of healthy eating, physical activity and obesity prevention policies, practices or programmes within childcare services | Wrong intervention |
| 91 | Ziso 2022 | Increasing Access to Healthy Foods through Improving Food Environment: A Review of Mixed Methods Intervention Studies with Residents of Low-Income Communities. | Wrong intervention |
| 92 | Zlatevska 2018 | Mandatory calorie disclosure: A comprehensive analysis of its effect on consumers and retailers | Wrong study design |

## Table. S5: Characteristics of studies awaiting classification (n=3)

| **Study ID** | **Title** | **Aim** | **Population** | **Intervention** | **Comparator** | **Outcome** | **Time frame** | **Study design** |
| --- | --- | --- | --- | --- | --- | --- | --- | --- |
| McNeill 2010 (conference abstract with no associated journal publication) ^93^ | Using evidence to prioritise areas for public health actions for tackling childhood overweight | To use evidence from systematic reviews of environmental influences on childhood overweight to prioritise areas for development of public health interventions. | Children up to 8 years | 1.Nutrient menu labelling 2. Portion size of manufactured foods and of restaurant and cafeteria items | NR | Ranking of environmental factors to identify those which should be prioritised for future public health interventions. | NR | Interventional and longitudinal studies |
| Sanz-Valero 2015 (conference abstract with no associated journal publication) ^94^ | Population interventions in food labelling regarding nutritional composition | To review systematically the literature around the interventions made by labelling in the prevention of obesity, information on the salt content, genetically modified food (GM) and the fat content of foods. | NR | 1.Food labelling with nutrient information to reduce salt intake  2. FL to reduce salt intake | NR | Food consumption (not observed in fast food restaurants) | NR | NR |
| Stran 2013 (journal publication, could not be accessed) ^95^ | Mandating nutrient menu labelling in restaurants: potential public health benefits | To examine the potential health benefits of nutrient menu labelling in restaurants, the progress of this legislation and to provide results regarding the implementation of these policies. | Adults | Nutrient menu labelling in restaurants | NR | Nutrition knowledge and food behaviours | NR | NR |

## Table. S6: Characteristics of included protocols (n=9)

| **Study ID** | **Title** | **Population and settings** | **Intervention** | **Comparator** | **Outcome** | **Study design** |
| --- | --- | --- | --- | --- | --- | --- |
| Almeida 2021 ^96^ | Promotion of fruit and vegetables’ choice, purchase or consumption: a systematic review of nudge interventions | Children, adolescents or adults. | Intervention: products’ proprieties (functionality, presentation, size or information) | 1. Intervention/exposure to another intervention or a non-exposed control group   2. Comparator group - before/ after | NA | Quantitative studies- experimental, quasi-experimental, studies with pre-post designs, randomized cross-over controlled trials and non-randomized cross-over controlled trials. |
| Al-Tamimi 2021 ^97^ | Food Environment Interventions to Improve the Dietary Behaviour of Hospital Staff: A Systematic Literature Review | Includes adult employees working within a hospital, excludes customers purchasing food outside of a hospital cafeteria setting.  Studies conducted within the retail food environment of a hospital. | Change to the food environment within hospitals that increase accessibility to healthy foods. These interventions may include the introduction of choice architecture, point-of purchase prompts or price incentives/ disincentives. | The control will be hospital environments where the intervention aimed at changing the food environment has not been introduced | Changes in eating patterns and food purchasing behaviour | Intervention studies such as Randomised/Cluster Randomised/ Quasi Randomised Control Trial, Time Series, Controlled, Observational, Pre-post. |
| Boelsen-Robinson 2021 ^98^ | Effectiveness outcomes of local government actions in food environments: a systematic review | Any food environment in high-income countries (as assessed by the World Bank). Food environment includes the community (type and location of food outlets, accessibility), organizational (home, school, work, other), consumer (availability, price, promotion, placement and nutrition information) and the information environment (media, advertising). | Nutrition interventions and policies where local governments are explicitly mentioned as being involved in the coordination, execution, design, evaluation, funding, and/or implementation of the action on food environments. | None required, will be included if available. | 1. Change in purchasing or consumption; 2. Change in purchasing or consumption; Measures of business outcomes 3. Change in population prevalence of nutrition or NCDs. 4. Effectiveness of changes to the food environment | Qualitative, quantitative and mixed method studies |
| Hart 2022 ^99^ | A Systematic Review of food environment interventions implemented in tertiary education settings | Students and staff at tertiary education settings. | Interventions that target the 4P’s of marketing (placement, product (including labelling), promotion, and price). | None or as described (e.g., isolation of food environmental modifications, closely matched nonadjacent sites, measured absence of intervention mechanisms). | Increase in sales/purchases of healthy foods and drinks  Increase in self-reported intake of healthy foods and drinks | Randomised controlled trials, pre and post (quasi-experimental) intervention studies, cross-sectional studies |
| Kathiresan 2022 ^100^ | Effect of nutritional labelling of restaurant menus on consumer food choices | Adolescents (over 10 years of age) and adults, any gender | Nutritional labelling | Comparator group -other interventions Comparator group - no intervention | 1. Mean Calories (kCal), Mean total salt content (g), Mean total trans-fat content (g), Proportion of healthier items (%) Proportion of high refined sugar items (%) 2. Means total cost of the items ordered. | 1.All interventional studies (randomized or non-randomized; individual or cluster randomized) with a control arm.  2.Observational studies (prospective and retrospective cohort) are included. |
| Polden 2022 ^101^ | Does the impact of calorie (energy) labelling of food and drink products on consumer behaviour differ by socioeconomic status? | Studies of human participants (adults and children) are eligible for inclusion, Restaurant, Laboratory, Online, | Studies are required to have an ‘experimental’ condition (including ‘natural’ and quasi-experiments) or trial arm in which energy content information (i.e., number of kcal) for food and/or drink products was provided to participants at point of food/drink selection or consumption. | No interventions | Energy Content | 1. Experimental studies using between-subjects and/or within-subject designs. 2. Longitudinal study designs using pre-post test designs are eligible for inclusion. |
| Schruff-Lim 2020 ^102^ | The effect of nutrition label+ interventions on the healthiness of food and non-alcoholic beverage choices | Lab and field setting. No specific exclusion criteria | Intervention of interest are nutrition label+ interventions. Nutrition label+ interventions are defined as multicomponent interventions with both a nutrition label and a plus component. | Comparator group -other interventions Comparator group - no intervention | Selection and Purchase | 1. Experimental design including case/control or before/after studies.  2. Studies using a multi-arm design |
| Schulte 2019 ^103^ | Choice architecture interventions (CAI) promoting sustained healthier food choice and consumption by students in a secondary school setting: a systematic review of intervention studies | Healthy adolescents aged 10 or higher. secondary school setting | Choice architecture interventions (CAI) | Comparator group - before/ after | Changes in food choice and consumption | Randomized controlled trials, pilot studies and quasi-experimental studies except Qualitative studies |
| Shafique 2022 ^104^ | Factors influencing the food environment in urban informal settlements in South Asia: a systematic review | Urban population (city, municipality, town, etc.) and informal settlements (e.g., slums) in South Asian countries (Afghanistan, Bangladesh, Bhutan, India, Maldives, Nepal, Pakistan, and Sri-Lanka). | Factors influencing any one or multiple dimensions of the food environment will be considered; for example – food provision, food retail, food composition, marketing, food labeling etc. | Since the review will mainly focus on the identification of factors that influence the food environment for the urban poor in South Asia, no criteria against control groups will be applied in this systematic review. | 1. Any changes/effects in the dimensions of the food environment. 2. Personal domain of the food environment- dimensions of food accessibility, affordability and desirability . 3. factors for adolescents /school-going children exposed to unhealthy food environments/ food choices  4. Any social and gender dimension of the food environment | Quantitative, qualitative, and mixed-method studies |

## Table. S7: Characteristics of excluded protocols (n=23)

| **S.No.** | **Study ID** | **Title** | **Reason for exclusion** |
| --- | --- | --- | --- |
| 1 | Bailey 2018 | Stakeholder perspectives of the facilitators and barriers to the implementation of school food and beverage innovations: A mixed-method study systematic review | Wrong intervention |
| 2 | Bandy 2022 | The impact and feasibility of healthy eating interventions conducted in small, local business settings: A systematic review | Wrong intervention |
| 3 | Bocardi 2021 | Hospital food environment and food consumption and anthropometric data of workers and patients: a systematic review | Review discontinued |
| 4 | Braune 2021 | A systematic review of the interpersonal determinants of diet quality and eating behaviours in young people | Wrong intervention |
| 5 | Dussort 2017 | A systematic review of the short- and long-term impacts of nudging on healthy food choices and intake in adults | Wrong intervention |
| 6 | Eulert 2022 | Behavioural interventions (Nudging strategies) to facilitate plant-based food consumption. Systematic Review | Wrong intervention |
| 7 | Ferreira 2019 | Food environment and relation to diet and health in children and adolescents: a systematic review | Wrong intervention |
| 8 | Gholambareshi 2020 | Systematic review of nutritional interventions performed in the Iranian school setting | Wrong intervention |
| 9 | Gupta 2021 | To describe food environment settings with multiple independent, competitive food outlets and identify interventions to promote healthy and environmentally sustainable eating in such settings | Wrong intervention |
| 10 | Haynes 2016 | The effect of beverage positioning in retail environments on sugar-sweetened beverage purchase and consumption | Wrong intervention |
| 11 | Kapoor 2022 | The UK food environment: a systematic review of characteristics and associations with diet and health | Wrong intervention |
| 12 | King 2018 | Retail food environment surrounding schools and childhood obesity: a systematic review | Wrong intervention |
| 13 | Knai 2020 | Food environment and relation to diet and health in children and adolescents: a systematic review | Wrong study design |
| 14 | Misra 2021 | Food environment interventions promoting healthy and/or sustainable eating: a systematic review | Wrong intervention |
| 15 | Neve 2020 | How does the food environment influence people engaged in weight management? A systematic review and thematic synthesis of the qualitative literature | Review discontinued |
| 16 | Nguyen 2018 | The effectiveness of community-based initiatives to increase fruit and vegetable intake in families: a systematic literature review | Wrong intervention |
| 17 | Osei 2022 | Influence of the school food environment on the nutritional status of school age children between 6 and 12 years in Sub-Saharan Africa. | Wrong intervention |
| 18 | Penney 2014 | Local food environment interventions to improve healthy food choice in adults: using a systematic realist synthesis to address the program theory gap | Review discontinued |
| 19 | Pineda 2018 | The food environment and its association with obesity: a systematic review and meta-analysis of observational studies | Wrong intervention |
| 20 | Ramírez 2018 | Environmental interventions in and around schools to reduce the consumption of sugar-sweetened beverages (SSBs) in children and adolescents: a systematic review of the literature | Wrong intervention |
| 21 | Shaw 2019 | A systematic review describing the role of the community and consumer nutrition environment on adolescents’ dietary and food purchasing behaviours | Wrong intervention |
| 22 | Vilela 2020 | Interventions on the school food environment and noncommunicable chronic diseases in children and adolescents: a systematic review and meta-analysis | Wrong intervention |
| 23 | Ziegler 2018 | Effectiveness of food environment interventions on changing diet quality with adolescents in Canada, Australia, New Zealand, UK, and United States: a systematic review | Wrong intervention |

## Table. S8- Characteristics of included primary studies (n=24)

| **S.No.** | **Study ID** | **Title** | **Population and settings** | **Intervention** | **Comparator** | **Outcome** | **Study design** |
| --- | --- | --- | --- | --- | --- | --- | --- |
| 1 | Anderson, 2015 ^105^ | Is level of intuitive eating associated with plate size effects? | College students | Intuitive eating after four hours of daytime fasting in small plate vs large plate | Comparing different plate sizes (small vs. large plate) | Greater food consumption | Randomized controlled trial |
| 2 | Ayaz, 2016 ^106^ | Effect of plate size on meal energy intake in normal-weight women | Female participants aged 19-25 years from Hacettepe University and the surrounding community | Offering lunch in different diameters of plate including small (19 cm), medium (23 cm), or large (28 cm) | Comparing different plate sizes (small (19 cm) vs medium (23 cm) vs large (28 cm) diameter plate) | Energy and micronutrient intake | Randomised cross-over study |
| 3 | Crickerd, 2017 ^107^ | Texture-based differences in eating rate reduce the impact of increased energy density and large portions on meal size in adults | Women who are healthy regular breakfast consumers | i. Thin porridge (140 g/min) compared with thick porridge (77 g/min)] ii. Increase in portion size by 50% increase (150%) | i. Comparing different food textures (thin vs thick porridge) ii. Comparing different portion sizes (100% vs 150%) | Combination of energy-density dilution, smaller portions, and natural variations in food texture to design meals that promote reductions in energy intake while maintaining satiety | Randomized controlled trial |
| 4 | Cullen, 2005 ^108^ | Texas school food policy changes related to middle school a la carte/snack bar foods: potential savings in kilocalories | Middle schools in the Houston area | Substituting reduced portion sizes for the large servings | Comparing different portions sizes (reduced vs. large servings) | Per student, about 111 kcal per day was purchased; 47 kcal per day was saved when reduced portion sizes were substituted for the large servings | Observational study |
| 5 | Gavelle, 2019 ^31^ | The initial dietary pattern should be considered when changing protein food portion sizes to increase nutrient adequacy in French adults | Adults aged >65 years and under- and over reporters | Manipulating the intake of foods already consumed and introducing foods consumed by >10% of individuals with the same protein pattern | Before and after comparison | Dietary protein pattern | Observational study |
| 6 | Goffe, 2018 ^109^ | Feasibility of working with a wholesale supplier to co-design and test acceptability of an intervention to promote smaller portions: an uncontrolled before-and-after study in British Fish & Chip shops | Owner, manager and costumer in northern England | Box packaging to serve smaller portions, promotional posters and business incentives | Before and after comparison | Acceptability of, an intervention to promote smaller portions | Quasi-experimental study |
| 7 | Haynes, 2020a ^110^ | Reductions to main meal portion sizes reduce daily energy intake regardless of perceived normality of portion size: a 5 day cross-over laboratory experiment | Individuals aged between 18-60 yrs and BMI 22.5 to 32.5 in a laboratory setting | Different portion sizes of the main meal component including 'large-normal' (747 kcal), ‘small-normal’(543 kcal) and 'smaller than normal’ (339 kcal) | Comparing three portion sizes including 'smaller than normal', 'small normal' and 'larger than normal' | Daily energy intake | Randomized cross-over study |
| 8 | Haynes, 2020b ^111^ | Portion size normality and additional within-meal food intake: two crossover laboratory experiments | University staff and students and in the local community | Different-sized lunchtime portions were served on three occasions | Comparing three portion sizes including 'smaller than normal', 'small normal' and 'larger than normal' | Reduction in total meal intake | Randomized cross-over study |
| 9 | Levitsky, 2004 ^112^ | The more food young adults are served, the more they overeat | Undergraduate students and staff | Different serving sizes in a buffet lunch offered on Monday, Wednesday, and Friday including 100%, 125%, or 150% of the amount of food they had consumed the previous week | Before and after comparison | i. Energy intake ii. Body weight | Randomized cross-over study |
| 10 | Levitsky, 2011 ^113^ | Losing weight without dieting. Use of commercial foods as meal replacements for lunch produces an extended energy deficit | Volunteers from the Cornell University campus | Meals and snacks served from Monday to Friday for five consecutive weeks.1st week: Food from a buffet where each food was weighed before and after eating.2nd & 3rd weeks: Lunch by choosing one food from a selection of six commercially available portion-controlled foods.4th & 5th week: The conditions were reversed for the two groups. | Before and after comparison | i. Daily energy intake ii. Weight loss | Randomized cross-over study |
| 11 | Libotte, 2014 ^114^ | The influence of plate size on meal composition: Literature review and experiment | Males and females | Individually invited to serve themselves a lunch from a buffet containing 55 replica food items in a standard-size plate (27 cm) or a large plate (32 cm) | Comparing different plate sizes (standardized vs. large plate) | i. Energy intake in order to promote weight loss. ii. vegetable consumption | Randomised controlled trial |
| 12 | Masterson, 2022 ^115^ | Perceived portions of foods affect the amount selected within an immersive virtual food environment | Female participants aged 28 to 40 years | Executing virtual reality buffet and laboratory meals in standard and large conditions | Comparing different portion sizes (standard vs. large conditions) | Participants selected more total food and energy in the large condition compared to the standard condition | Randomized cross-over study |
| 13 | Myers, 2019 ^116^ | Portion size influences intake in Samburu Kenyan people not exposed to the western obesogenic environment | Samburu people | Individual serving bowl containing 1.4 or 2.3 kg of a familiar bean and maize stew | Comparing different portion sizes | Food intake | Randomized cross-over study |
| 14 | Raghoebar, 2019 ^117^ | Served portion sizes affect later food intake through social consumption norms | UK adults via the online survey platform | Visual exposure to smaller/larger food portions and consumption | Comparing different portion sizes (smaller vs. larger food portion) | Food intake | Quasi-experimental study |
| 15 | Reinders, 2020 ^118^ | Portioning meat and vegetables in four different out of home settings: A win-win for guests, chefs and the planet | Diners of the privately-owned, middle-sized, French cuisine a-la-carte restaurant | Reduction of fish/meat portion by 8-16% | Before and after comparisons Vegetable consumption vs. fish/meat consumption | 1. Decrease in fish/meat consumption 2. Increase in vegetable consumption | Quasi-experimental study |
| 16 | Reynolds, 2021 ^119^ | Impact of decreasing the proportion of higher energy foods and reducing portion sizes on food purchased in worksite cafeterias: A stepped-wedge randomised controlled trial | Employees mostly in manual occupation in cafeteria setting | Replacing some higher energy foods with lower energy foods (availability) and reducing the portion size of some higher energy foods | Before and after and comparison of two interventions | Energy(kcal) purchased per day | Randomized controlled trial (stepped wedge design) |
| 17 | Robinson, 2019 ^120^ | When a portion becomes a norm: Exposure to a smaller vs. larger portion of food affects later food intake | Female participants | Smaller and larger biscuits stick | Comparing different serving sizes (smaller vs. larger biscuit sticks) | Food intake | Randomized controlled trial |
| 18 | Rolls, 2004 ^121^ | Increasing the portion size of a sandwich increases energy intake | Young adults from a university community | Different serving sizes of a deli-style sandwich including 6, 8, 10, or 12 inches in lunch served in a lab once a week for four weeks | Before and after comparison | Energy intake | Quasi-experimental study |
| 19 | Rolls, 2007 ^122^ | Using a smaller plate did not reduce energy intake at meals | Adults aged 20–45 years from a university community | i. Three different plate sizes to serve main course including 17, 22 and 26 cm ii. Equal amount of food presented on each of the two larger plates iii. Used each of the three plates and selected from a buffet of five foods matched for energy density | Comparing different plate sizes | Energy intake | Randomised cross-over study |
| 20 | Sheen, 2018 ^123^ | Plate-clearing tendencies and portion size are independently associated with main meal food intake in women: A laboratory study | Female participants (self-reported habitual plate clearers or non-plate clearers) | Different portion sizes of pasta for lunch including ‘normal’ (500g) and ‘large’ (1000g) | Comparing different portion sizes (normal (500g) vs. large (1000gm)) in plate clearers vs. non-plate clearers | Increasing the portion size of the lunchtime meal increased food intake, although the tendency for a larger portion size to increase food intake was observed irrespective of participant plate-clearing tendencies | Randomised controlled trial |
| 21 | Vieux, 2013 ^124^ | Dietary standards for school catering in France: Serving moderate quantities to improve dietary quality without increasing the food-related cost of meals | Primary schools | Portion size control | Before and after comparison | Food-related cost of meals | Quasi-experimental study |
| 22 | Wansink, 2013 ^125^ | Portion size me: Plate-size induced consumption norms and win-win solutions for reducing food intake and waste | Students aged between 18 and 28 with an average age of 20 | Different serving sizes of cereals (cheerios, corn, pops and cornflakes) served in six set of bowls | Comparing different serving sizes | i. Visual fill- level size of one’s consumption norm relative to the level they perceive as generally appropriate ii. Influence of norms on serving and consumption in a natural eating environment | Quasi-experimental study |
| 23 | Yip, 2013 ^126^ | Using a smaller dining plate does not suppress food intake from a buffet lunch meal in overweight, unrestrained women | Overweight/obese women from Auckland, New Zealand | Eat lunch in a small (19.5 cm) or a large (26.5 cm) diameter dining plate | Comparing different plate sizes (small vs. large plate size) | Food intake | Randomised controlled trial |
| 24 | Zuraikat, 2018 ^127^ | Doggy bags and downsizing: Packaging uneaten food to go after a meal attenuates the portion size effect in women | Women aged between 18 and 60 years | Portion size increased to 125%, 150%, and 175% of the baseline amount (to-go-group) | Average intake of a similar baseline meal (100%) consumed by women in a previous study (Control group) | Packaging uneaten food after a meal could be an effective strategy to reduce overconsumption from large portions | Randomised cross-over study |

## Table. S9- Characteristics of excluded primary studies (n=16)

| **S. No.** | **Study ID** | **Title** | **Reason for the exclusion** |
| --- | --- | --- | --- |
| 1 | Blackham 2018 | Challenges to improve the nutritional quality of foods served by small independent takeaway outlets | Wrong study design |
| 2 | Blackham 2022 | Takeaway food consumers (in Liverpool) are receptive to the idea of reformulated healthier versions of takeaway foods | Wrong intervention |
| 3 | Blum 2007 | Implementation of low-fat, low-sugar (LFLS), and portion-controlled nutrition guidelines in competitive food venues of maine public high schools: Research article | Wrong intervention (compliance with LFLS intervention) |
| 4 | Boer 2014 | "Meatless days" or "less but better"? Exploring strategies to adapt Western meat consumption to health and sustainability challenges | Wrong intervention |
| 5 | Fuster 2020 | Ethnic Restaurant Nutrition Environments and Cardiovascular Health: Examining Hispanic Caribbean Restaurants in New York City | Wrong intervention |
| 6 | Lasschuijt 2019 | Unaware of the amount consumed: Systematic error in estimating food- and drink intake | Wrong setting |
| 7 | Lock 2016 | Contextual and environmental influences on reported dietary energy intake at evening eating occasions | Wrong setting |
| 8 | Moore 2020 | Irish children's food and beverage portion sizes: A qualitative study of parents' views and practices | Wrong study design |
| 9 | Ofei 2015 | Effect of meal portion size choice on plate waste generation among patients with different nutritional status. An investigation using Dietary Intake Monitoring System (DIMS) | Wrong intervention |
| 10 | Reale 2018 | The Feasibility and Acceptability of Two Methods of Snack Portion Control in United Kingdom (UK) Preschool Children: Reduction and Replacement | Wrong setting |
| 11 | Rodrigues 2012 | Overweight/obesity is associated with food choices related to rice and beans, colors of salads, and portion size among consumers at a restaurant serving buffet-by-weight in Brazil | Wrong intervention |
| 12 | Schwartz 2010 | The relative influence of calorie labeling and behavioral economic nudges in altering fast food choice | Wrong intervention |
| 13 | Seto 2016 | Models of Individual Dietary Behavior Based on Smartphone Data: The Influence of Routine, Physical Activity, Emotion, and Food Environment | Wrong intervention |
| 14 | Sheen 2020 | Food waste concerns, eating behaviour and body weight. | Wrong intervention |
| 15 | Vargas-Alvarez 2022 | Development and validation of a new methodological platform to measure behavioral, cognitive, and physiological responses to food interventions in real time | Wrong intervention (methodological study) |
| 16 | Young 2015 | Behavioral mediators of weight loss in the SHED-IT community randomized controlled trial for overweight and obese men | Wrong intervention |

## Figure. S1 Time trends of the included reviews on menu labeling and portion size control (n = 69)


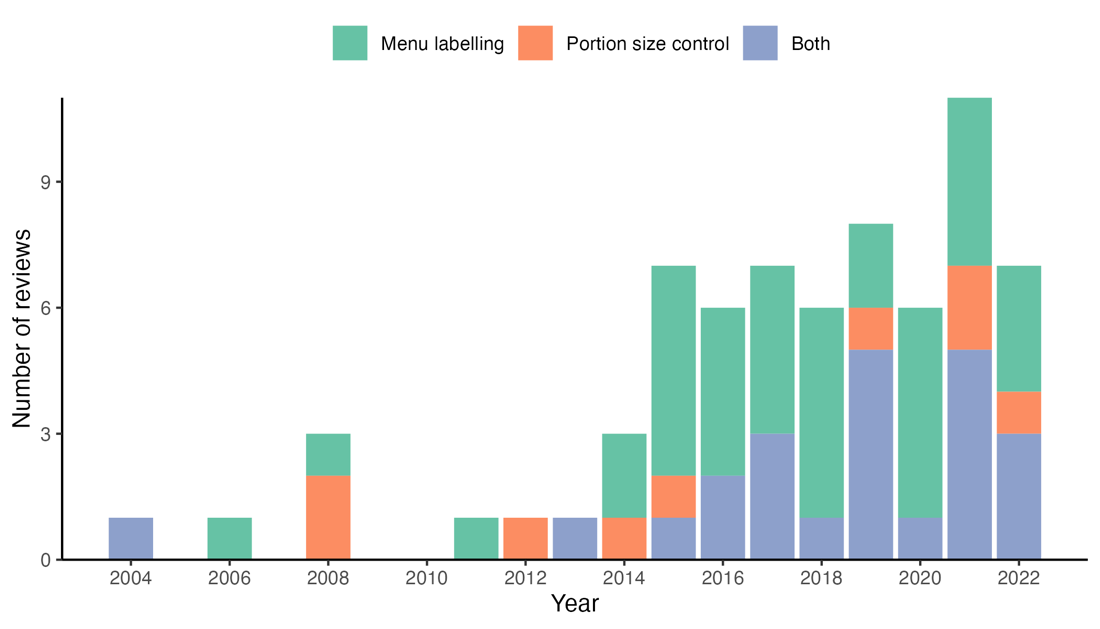


## Figure. S2. Geographical distribution of the included reviews on menu labeling and portion size control in out-of-home food environment (n=69)


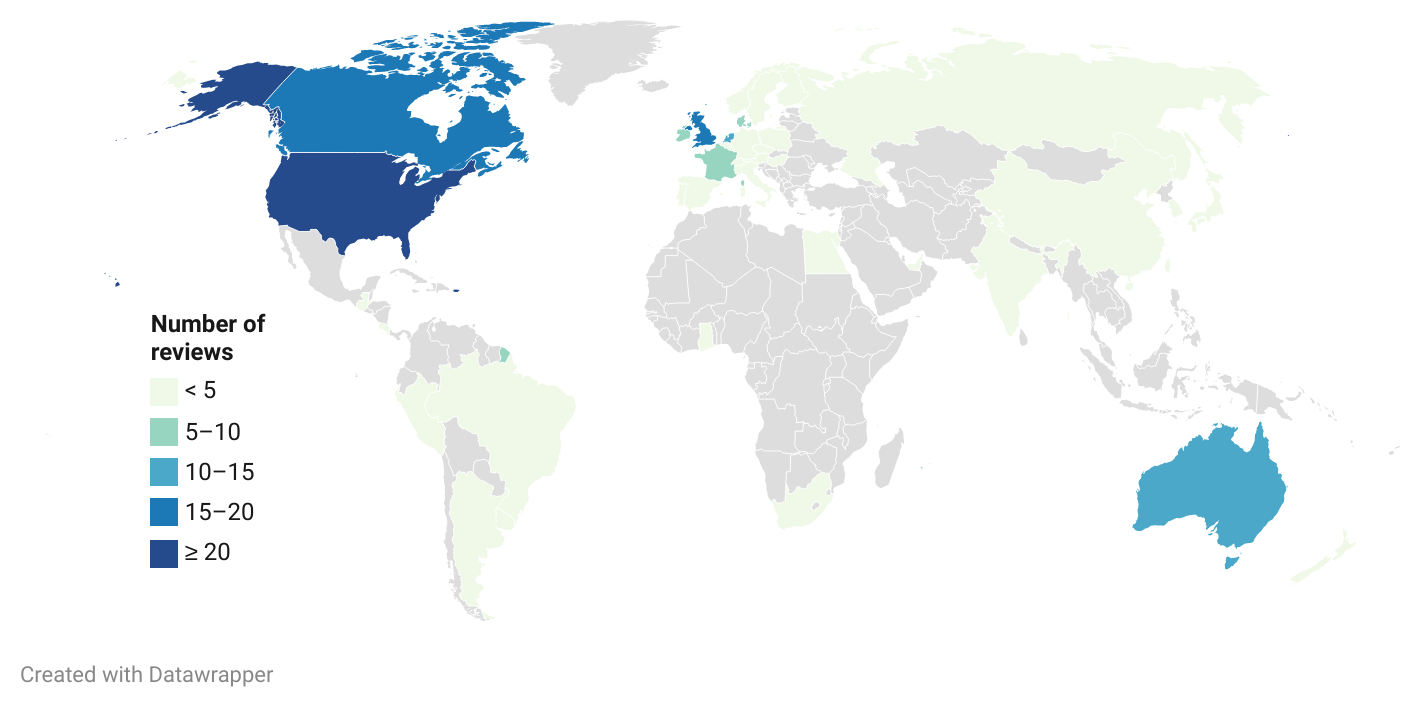

Supplement: Supplementary file 1 — Supporting information. [file CESM-2-e12039-s001.docx]
